# Soluble endoglin induces trophoblast apoptosis and dysfunction via the CD39-ATP signaling axis: a potential pathogenic mechanism of preeclampsia

**DOI:** 10.1186/s12967-026-08759-x

**Published:** 2026-08-10

**Authors:** Yu Wang, Yiyang Qu, Dongjie Wang, Yiming Zhang, Yingya Li, Yanping Jia, Kunming Li, Liyan Duan

**Affiliations:** 1 Tongji University School of Medicine, Shanghai Tenth People’s Hospital of Tongji University, Shanghai, 200072 China; 2 Hubei University of Chinese Medicine, Wuhan, 430000 China; 3 Shanghai Tenth People’s Hospital of Tongji University, No.301, Middle Yanchang Road, Shanghai, 200072 China

**Keywords:** Soluble endoglin, Preeclampsia, Trophoblast, Apoptosis, CD39, Extracellular ATP

## Abstract

**Objective:**

Excessive trophoblast apoptosis and dysfunction are critical events in the pathogenesis of preeclampsia (PE). Soluble endoglin (sENG) is elevated in PE; however, its direct role in regulating trophoblast biology remains incompletely understood. In this study, we investigated whether sENG promotes trophoblast apoptosis and impairs trophoblast function, and further explored the underlying molecular mechanisms.

**Methods:**

Placenta-targeted sENG overexpression was induced in pregnant mice via an AAV6 vector driven by the FLT1 promoter, and PE-like phenotypes including hypertension and proteinuria were evaluated. Transforming growth factor-β (TGF-β)-Smad2/3 signaling, CD39 expression, extracellular ATP (eATP) levels, and P2X7 receptor (P2X7R) expression were analyzed in placental tissues and maternal serum. In vitro, HTR-8/SVneo trophoblasts were treated with recombinant sENG, and trophoblast biological functions and signaling protein expression were assessed by CCK-8, wound healing, Transwell, immunofluorescence, and western blotting. Rescue experiments were performed using exogenous TGF-β1 and a P2 × 7R inhibitor. Key signaling changes were further validated in placental tissues from patients with PE and healthy controls.

**Results:**

In mice, sENG overexpression triggered hypertension, proteinuria, fetal growth restriction, and placental apoptosis, accompanied by inhibition of the TGF-β1-Smad2/3-CD39 pathway. In vitro, sENG induced dose-dependent apoptosis by suppressing Smad2/3 phosphorylation, downregulating CD39, and elevating eATP levels; these effects were reversed by exogenous TGF-β1. BzATP-mediated P2X7R activation contributed to trophoblast dysfunction and apoptosis. In human PE placentas, reduced phosphorylation of Smad2/3 and decreased CD39 expression, together with increased P2X7R expression, were associated with enhanced apoptotic signaling.

**Conclusion:**

sENG induces trophoblast apoptosis and dysfunction through the TGF-β-Smad2/3-CD39-eATP-P2 × 7R signaling axis, contributing to PE pathogenesis and representing a promising molecular candidate for future therapeutic research into PE.

**Graphical Abstract:**

**Figure:**
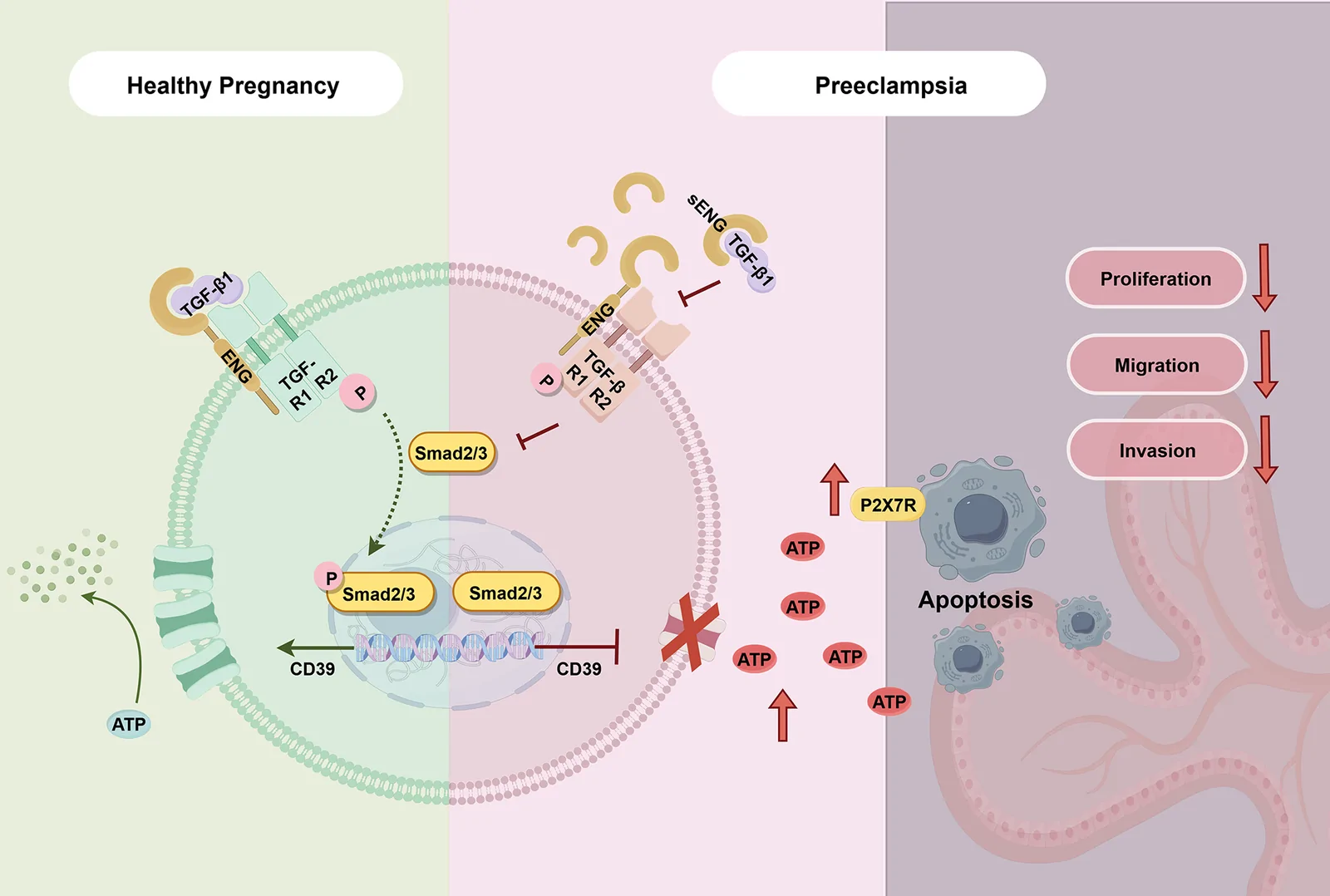

**Supplementary Information:**

The online version contains supplementary material available at https://doi.org/10.1186/s12967-026-08759-x.

## Introduction

Preeclampsia (PE) is a progressive, multisystem disorder unique to pregnancy. Globally, it affects 5–7% of pregnant women, accounting for over 70,000 maternal deaths and 500,000 fetal deaths annually [1]. The main clinical manifestations range from mild hypertension and proteinuria to severe renal impairment, hepatic dysfunction, retinopathy, and neurological complications. The severity and pace of progression vary substantially among patients, posing major challenges for clinical diagnosis and management [2]. It is well established that the pathogenesis of PE is rooted in abnormal placental function [3], endothelial dysfunction, and vasoconstriction, culminating in systemic multiple organ dysfunction [4]. Although the pathogenesis and clinical management of PE have been extensively investigated worldwide, there is still a lack of established, specific, and effective treatments. Available interventions are mainly aimed at relieving symptoms and preventing complications, and the only definitive approach identified to date is timely delivery [5]. Thus, pathogenesis-based early diagnosis and effective intervention represent critical steps toward better outcomes in PE. These efforts may also facilitate the development of novel therapeutic strategies for PE.

Apoptosis, a tightly regulated mode of programmed cell death, is a ubiquitous biological phenomenon that eliminates damaged or nonviable cells, thereby protecting the organism against potential pathological changes [6]. In normal pregnancy, placental apoptosis is in a dynamic balance to maintain normal development and function. In PE, this homeostatic balance is disturbed, as oxidative stress, inflammatory signaling, and placental ischemia–hypoxia can aberrantly activate apoptotic pathways in placental cells [7, 8]. For instance, excessive reactive oxygen species (ROS) may cause cellular DNA damage, activating intrinsic apoptotic pathways, amplifying caspase-dependent cascades, and leading to cell apoptosis [9]. Additionally, abnormal elevation of inflammatory factors, including tumor necrosis factor‑alpha (TNF‑α), can activate cell-surface death receptors via the extrinsic apoptotic pathway, thereby inducing cellular apoptosis [10]. Aberrant apoptosis compromises placental architecture and function, leading to defective placental vascular remodeling and diminished trophoblast invasion, culminating in the clinical manifestations of PE. Unraveling the molecular regulatory networks governing placental apoptosis thus holds substantial implications for refining diagnostic precision and advancing therapeutic interventions for PE.

Previous studies have found that plasma ATP levels are approximately 2.5-fold higher in patients with PE than in women with normal pregnancies [11]. Extracellular ATP (eATP) acts as a damage-associated molecular pattern (DAMP) that is released during cell activation, stress, injury, or necrosis, thereby triggering inflammatory responses [12]. Therefore, eATP may contribute to the pathogenesis of PE. ATP infusion in pregnant rats induces PE-like phenotypes, including proteinuria, altered systemic immune responses, and placental ischemia, which are not observed in non-pregnant controls [13]. However, the precise mechanism by which ATP exerts its effects remains incompletely understood. Nevertheless, as mentioned above, it is likely that ATP directly influences trophoblast function and endothelial progenitor cell-mediated vascular repair. Under physiological conditions, eATP concentrations are maintained at low levels [14], since the abnormal release of ATP can be sequentially hydrolyzed by specific nucleotide enzymes such as CD39. CD39, also known as ectonucleoside triphosphate diphosphohydrolase 1 (ENTPD1), belongs to the NTPDase family and catalyzes the sequential hydrolysis of ATP to ADP and then to AMP, is involved in multiple disease states, including cancer, autoimmune disorders, and inflammatory diseases [15]. In colorectal cancer cells, CD39 modulates cell invasion and apoptosis [16]. Concurrently, the expression of CD39 has been shown to confer protective effects in murine models of antiphospholipid antibody-induced miscarriage and PE [17, 18]. Thus, it is reasonable to conclude that CD39-dependent regulation of eATP levels is a pivotal mechanism governing cellular stress responses and apoptotic cell death, with profound implications for the development of placental dysfunction in PE.

Soluble endoglin (sENG) is the proteolytic cleavage product of the extracellular domain of endoglin (ENG), a co-receptor of the transforming growth factor β (TGF-β) superfamily. Our preliminary studies revealed that plasma sENG levels were significantly elevated in patients with PE; following therapeutic plasma exchange, levels of sENG, placental inflammation and apoptosis were reduced [19]. sENG is widely recognized for its antiangiogenic properties, contributing to endothelial dysfunction and the pathogenesis of cardiovascular diseases [20, 21]. Upon release into the circulation, sENG acts as a decoy receptor, sequestering ligands such as TGF-β1, bone morphogenetic protein (BMP)-9, and BMP-10. This sequestration reduces the bioavailability of TGF-β1, thereby disrupting canonical TGF-β1 signaling homeostasis [22]. Studies have demonstrated that sENG plays an important role in intercellular communication between NK cells and trophoblasts by signaling through ALK5‑Smad2/3 [23]. Raphael S Peres et al. reported that TGF‑β increases CD39 expression on regulatory T (Treg) cells via activation of TGFBRII/TGFBRI, Smad2, and the transcription factor CREB [24]. In addition, accumulating evidence indicates that sENG not only contributes to endothelial dysfunction in the maternal peripheral vasculature in PE but also has an adverse impact on placental function [25]. Despite these observations, the precise mechanisms by which sENG regulates CD39 expression and impairs trophoblast biology remain poorly understood.

In summary, this study aimed to investigate the effects of elevated sENG levels on trophoblast function, analyze the specific mechanisms driving PE pathogenesis, and elucidate the role of sENG in regulating eATP through CD39 in this process. Our findings provide a theoretical foundation for a deeper understanding of the pathophysiological mechanisms underlying PE.

## Materials and methods

### Animal experiments

All animal experiments in the present study were performed following the ARRIVE 2.0 guidelines and were approved by the Institutional Animal Ethics Committee. Specific pathogen-free (SPF) C57BL/6J mice (6–8 weeks old) were purchased from a certified laboratory animal supplier and housed in an SPF-grade barrier facility under standardized environmental conditions: ambient temperature maintained at 22 ± 2 °C, relative humidity kept at 50 ± 5%, and a fixed 12 h light–dark photoperiod. All mice were provided with free access to sterilized standard rodent chow and autoclaved tap water ad libitum. Prior to experimental manipulation, all animals were allowd to acclimatize to the housing environment for 7 days.

Age- and body weight-matched virgin female and male C57BL/6J mice were randomly assigned for mating. Randomization was completed before cage rearrangement, and investigators remained blinded to group allocation during subsequent sample detection and data quantification to avoid subjective bias. Vaginal plugs of female mice were inspected at 08:00 on the next morning; the day when copulatory plugs were positively identified was defined as gestational day 0 (D0). Pregnant animals with abnormal body weight fluctuations, apparent illness, or miscarriage tendency were excluded. On D8, tail vein intravenous injection was conducted in gestation-matched pregnant dams: the experimental cohort received 5 × 10⁹ vg AAV6-mENG diluted in 100 µL sterile vehicle, whereas gestational stage-matched control pregnant mice were given an identical volume (100 µL) of AAV6-hrGFP empty vector solution. Non-invasive tail-cuff plethysmography was applied to sequentially detect maternal mean arterial pressure (MAP) from D8 to D19 after adequate equipment habituation. Following a 48 h adaptive training phase, eligible pregnant mice were individually placed into metabolic cages spanning D17.5–D18.5 to acquire 24 h continuous urine specimens, and urinary total protein content was determined via the Bradford colorimetric method. All terminal specimen harvests were performed on D19. In vivo viable fetal counts were determined immediately after laparotomy, followed by individual weighing of isolated fetuses and corresponding placentas.

### Cell culture and treatment

The human trophoblast cell line HTR-8/SVneo was kindly provided by the Shanghai First Maternity and Infant Hospital. These cells were cultured in RPMI-1640 medium supplemented with 10% fetal bovine serum and 1% penicillin-streptomycin, and incubated at 37 °C with 5% CO₂. BeWo cells were obtained from Fuheng Biotechnology Co., Ltd., and cultured at 37 °C with 5% CO₂. Ham’s F-12 medium supplemented with 10% fetal bovine serum and 1% penicillin-streptomycin mixture was used for cell culture. To ensure cell viability, the medium was changed every two days.

The exogenous reagents included: recombinant human sENG protein(TKC024, Tengke Biotech, Nanjing, China) with a concentration gradient of 0-160 ng/mL; human TGF-β1 recombinant protein(HY-P70543, MedChem Expression, New Jersey, USA) with a concentration of 5 ng/mL; ARL67156(1021868-83-6, MedChem Expression, New Jersey, USA), an inhibitor of CD39 with concentrations of 10µM and 20µM; ATP analog 2’,3’-O-(4-Benzoylbenzoyl) adenosine 5’-triphosphate(BzATP, HY-136254, MedChem Expression, New Jersey, USA) with a stimulation concentration gradient of 0-125µM; P2 × 7R inhibitor A-740,003 (HY-50697, MedChem Expression, New Jersey, USA) with a stimulation concentration of 10µM.

### Placental samples collection

Placental tissues were obtained from the Department of Obstetrics, Shanghai Tenth People’s Hospital of Tongji University, China, between 2024 and 2025. The respective ethics committee approved the study by obtaining consent forms (SHSY-IEC-6.0/25KY8/P01). Placental tissues were obtained at the time of vaginal delivery or cesarean section. Twenty pregnant women were recruited for this study, with 10 allocated to the PE group and 10 to the healthy control group. The baseline clinical characteristics of the two study groups are presented in Sect. 1 of the Supplementary Material.

Preeclampsia was diagnosed according to the International Society for the Study of Hypertension in Pregnancy(ISSHP) [26], and was defined as gestational hypertension accompanied by one or more new-onset following conditions at ≥ 20 weeks’ gestation: proteinuria; other maternal end-organ dysfunction including neurological and haematological complications, pulmonary oedema and liver involvement et al.; uteroplacental dysfunction. Full-thickness placental tissues were sampled from the central area of the placenta (within 2 cm of the umbilical cord insertion), spanning from the chorionic plate to the basal plate. Specimens with macroscopic infarcts, calcifications, or hematomas were excluded prior to the experiments. All tissues were washed twice with sterile phosphate-buffered saline (PBS) immediately after collection, frozen in liquid nitrogen, and stored at − 80 °C until RNA and protein samples were extracted.

### Hematoxylin and eosin (HE) and terminal deoxynucleotidyl transferase dUTP nick end labeling (TUNEL) staining

Human and mouse tissues were fixed overnight in 4% paraformaldehyde and processed into paraffin-embedded 5 μm sections. For HE staining, paraffin sections were deparaffinized and rehydrated, stained with hematoxylin for nuclear labeling, differentiated with hydrochloric acid alcohol, counterstained with eosin to visualize cytoplasmic structures, and finally sealed with neutral gum after dehydration and transparency to observe general placental morphological alterations under light microscopy. For TUNEL staining to detect apoptotic cells, dewaxed sections underwent antigen retrieval and endogenous peroxidase blocking. Sections were incubated with TUNEL reaction mixture according to the manufacturer’s instructions (A111-03, Vazyme, China), counterstained with DAPI to label all cell nuclei, and mounted for fluorescence microscopy analysis. The apoptotic level was quantitatively determined by calculating the percentage of TUNEL‑positive cells in each group.

### Immunofluorescence(IF) and immunohistochemistry (IHC)

HTR‑8/SVneo cells seeded on climbing slides were fixed with 4% paraformaldehyde for 30 min and permeabilized with 0.3% Triton X‑100 for 15 min. The paraffin sections were dewaxed, rehydrated, and subjected to antigen retrieval. For IHC, sections were quenched with 3% H₂O₂ for 15 min to block endogenous peroxidase. All samples were blocked with 5% BSA for 30 min, incubated with primary antibodies (Tables S2–S3) at 4 °C overnight, and then incubated with secondary antibodies for 1 h at room temperature, followed by DAPI (C1005, Beyotime, Shanghai, China) staining for IF and DAB staining and hematoxylin counterstaining for IHC. All images were captured using a Leica microscope, and the average optical density was quantified using ImageJ.

### Western blotting (WB)

Proteins from cells and tissues were extracted using a lysis buffer prepared with RIPA lysis buffer, protease inhibitor(P1005, Beyotime, Shanghai, China), and phosphatase inhibitor(P1045, Beyotime, Shanghai, China). Protein samples (20 µg) were separated by SDS–PAGE(10% or 12%) and transferred to PVDF membranes (10600029, Cytiva, USA). The membranes were blocked with 5% BSA in PBST for 1 h at room temperature and then incubated overnight at 4 °C with the primary antibody. The secondary antibody was incubated for 1 h at room temperature (for antibody information, see Table S4). An ECL chemiluminescence kit(P0018M, Beyotime, Shanghai, China) was used to detect the samples. Image J was used to perform densitometric analysis of single protein bands, after which the protein expression levels were normalized to those of GAPDH or β-tubulin. For samples separated on distinct western blot membranes, inter-blot calibration was first performed using a universal reference sample loaded onto every gel, followed by signal normalization and quantitative calculation. All blots containing samples from different experimental groups were imaged under identical exposure parameters to maintain consistent imaging conditions across groups.

### Cell counting kit-8 (CCK8) assay

To assess cell viability, HTR8/SVneo cells were seeded at a density of 5000 cells per 200 µl in 96 - well plates. The cells were stimulated with sENG at concentrations of 0-160 ng/mL or with BzATP at concentrations of 0-125 µM. A mixture of serum - free RPMI − 1640 medium and CCK8(C0038, Beyotime, Shanghai, China) at a ratio of 10:1 was added to the culture plates. After incubation in the dark for 2 h, absorbance was measured at 450 nm.

### Wound healing assay

HTR8/SVneo cell suspensions were seeded into 6‑well plates at 3 × 10⁵ cells per well. A uniform scratch was created in the confluent monolayer using a 200 µL pipette tip, and detached cells were rinsed away with PBS. Cells were incubated in medium containing sENG or BzATP (with or without A-740003). Images were acquired at 0 and 24 h using a Leica microscope and analyzed with ImageJ.

### Transwell experiment

Basement membrane matrix (BL1833, Biosharp, Beijing, China) was diluted 1:8 in serum-free medium, and 60 µL was coated onto Transwell upper chambers and gelled at 37 °C for 30 min. HTR8/SVneo cells (8 × 10⁴/500 µL) suspended in serum-free medium with sENG or BzATP (with or without A-740003) were seeded into upper chambers; 500 µL 10% FBS complete medium was added to the lower wells. After 24 h invasion, cells were fixed with paraformaldehyde and stained with crystal violet. Invasive cells were imaged via Leica inverted microscope and quantified with ImageJ.

### Quantitative real-time PCR

Total RNA was extracted using TRIzol (R0016, Beyotime, Shanghai, China) following the manufacturer’s protocol. cDNA was synthesized from 4 µg of total RNA in a 20 µl reaction volume using All-in-one RT EasyMix for qPCR(#22107, TOLOBIO, Nanjing, China). qPCR was performed using 2× SYBR qPCR Master Mix (#22204, TOLOBIO, Nanjing, China) on a LightCycler^®^ 96 Real-Time PCR System (Roche Diagnostics, Basel, Switzerland). GAPDH was used as an endogenous control for relative mRNA expression levels, which were calculated using the comparative Ct (2 − ΔΔCt) method. Primer sequences are provided in Table S5.

### Construction of CD39 overexpression plasmid and cell transfection

CD39 was overexpressed by using CD39 overexpression plasmid (CD39-OE) (Fenghui Biotechnology, Changsha, China). Vector represents the empty overexpression plasmid and serves as the negative control. HTR-8/SVneo cells seeded in 6-well plates were transfected at 70%–90% confluency using LipoMax™ transfection reagent (#32012, SUDGEN, China). Briefly, 2.5 µg of plasmid DNA and 6 µL of LipoMax reagent were separately diluted in serum-free medium. After incubation at room temperature, the two diluents were mixed thoroughly and applied to the cell cultures. Following 4–6 h of incubation with transfection complexes, the medium was replaced with serum-containing complete medium. Cells were harvested 48 h post-transfection for total protein extraction and subsequent western blot analysis of CD39 expression.

### Annexin V-FITC/PI staining assay

Following treatment with gradient concentrations of sENG, 1–3 × 10^6^ cells from each group were harvested and rinsed with pre-cooled PBS. Surface phosphatidylserine externalization was quantified using an Annexin V-fluorescein isothiocyanate (FITC)/Propidium Iodide (PI) apoptosis kit (A11015, MultiSciences, China) strictly following the manufacturer’s protocols. Stained cell samples were analyzed on a CytoFLEX flow cytometer (Beckman Coulter, Brea, CA, USA). Data were analyzed by using FlowJo software (BD Biosciences). The total apoptotic proportion for each group was calculated as the combined percentage of early apoptotic (Annexin V⁺PI⁻) and late apoptotic (Annexin V⁺PI⁺) cell populations.

### Enzyme-linked immunosorbent assay (ELISA)

The sENG levels in the serum of mouse models were detected according to the instructions provided by the reagent supplier. After terminating the reaction, the optical density (OD) values were measured at 450 nm. The four-parameter logistic method was used to fit the standard curve, and the sENG concentrations of the samples were calculated.

### eATP level measurement

The supernatant ATP values were normalized to total cell number. Briefly, HTR8 cells were seeded into six-well plates at a density of 3 × 10⁵ cells in 1.2 mL medium per well, and treatment as well as sample harvest were conducted at consistent time points among all experimental groups. Whole blood was collected from maternal mice via retro-orbital venipuncture into RNase-free microcentrifuge tubes. After 2 h of ambient temperature clotting, samples were spun at 3000 rpm for 15 min at 4 °C. The recovered serum was aliquoted and stored at − 80 °C, and all samples were measured together within one week of collection.

Both the supernatants of the cell cultures and the blood serum of mice were centrifuged at 12,000 rpm for 5 min at 4 °C before measurement. According to the instructions of the Addison Enzymatic ATP Assay Kit (ADS-W-A001-96), the reagents were added sequentially, and the absorbance was measured at 340 nm using an Infinite 200 PRO(Tecan, Switzerland). The corresponding ATP concentration was calculated according to the formula provided by the reagent supplier.

### Enzyme activity assay

Caspase-3 activity was quantified using a Caspase-3 activity assay kit (C1115, Beyotime, Shanghai, China), and all procedures were performed in accordance with the manufacturer’s instructions.

### Statistical analysis

For this study, sample size calculation was performed using GPower software. Baseline clinical data from healthy control and PE groups were analyzed with IBM SPSS Statistics. For in vivo animal experiments, raw data were averaged per litter; these litter-level mean values were defined as independent statistical units to eliminate statistical non-independence among pups originating from a single dam. All cellular experiments were performed with three independent biological replicates.

All statistical analyses were performed using GraphPad Prism. Definitive outliers were removed via the ROUT test with Q = 1%. The Shapiro-Wilk test was used to verify normality, and the F-test was applied to test homogeneity of variance (α = 0.05). Normally distributed data with homogeneous variances were compared using unpaired two-tailed t-test (two-group comparison) or analysis of variance (ANOVA) followed by Tukey’s or Dunnett’s post-hoc test according to experimental grouping design. Non-parametric statistical tests were adopted for datasets that violated normality or homoscedasticity criteria. All data are presented as mean ± SD. Statistical significance was set at two-tailed *P* < 0.05 (*), *P* < 0.01 (**), *P* < 0.001 (***), and *P* < 0.0001 (****).

Additional methods are provided in the Supplementary Material.

## Results

### Placenta-specific sENG overexpression recapitulates preeclamptic phenotypes and trophoblast apoptosis in mice

**Fig. 1:**
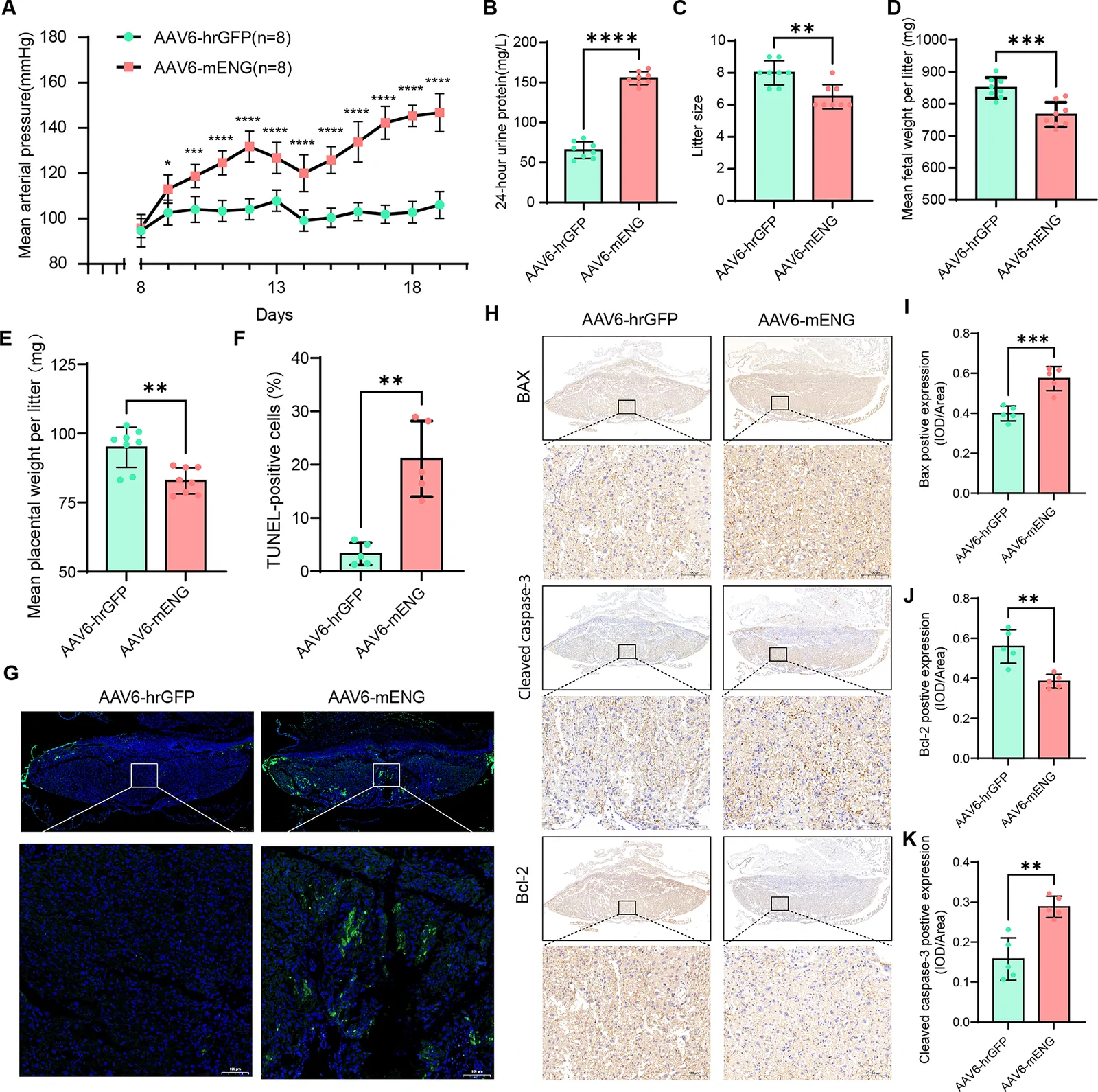
Overexpression of sENG in pregnant mice recapitulates PE-like phenotypes and promotes placental apoptosis. AAV6-hrGFP: Pregnant mice were injected with AAV6-hrGFP as the control group (*n* = 8). AAV6-mENG: Pregnant mice were injected with an equivalent volume of AAV6-mENG as the experimental group (*n* = 8). (**A**) Mean arterial blood pressure of both groups was measured from D8 to D19; (**B**) 24-hour urinary protein level of both groups at D17.5-D18.5; (**C**-**E**) The litter size, mean fetal weight and placental weight per litter of both groups; (**F**,**G**) Analysis and representative TUNEL staining of mouse placentas from both groups (*n* = 5); green: TUNEL; blue: DAPI; scale bar:100 μm; (**H**) Representative IHC staining of BAX, cleaved caspase-3 and Bcl-2 of placenta from both groups, scale bar:100 μm.; (**I**-**K**) Semi-quantitative analysis of BAX, cleaved caspase-3 and Bcl-2 expression in placenta (integrated optical density/area, *n* = 5). Statistical comparisons were performed via Šídák’s multiple comparisons test following two-way ANOVA (**A**), unpaired Student’s t-test (**B**, **D**, **E**), or non-parametric tests (**C**, **F**). Data were presented as the mean ± SEM. ** *P* < 0.01,***P < 0.001, ****P < 0.0001

To elucidate the in vivo pathogenic role of sENG, we generated a placenta-specific sENG-overexpressing mouse model (AAV6-mENG) using AAV6-hrGFP as a control. WB analysis of liver, kidney, lung, and placental tissues from mice in both groups was performed to verify the successful specific overexpression of mENG. The results revealed negligible ENG expression in extraplacental organs, while placental ENG abundance was markedly higher in the experimental group than in the control group(Fig. S1A-C). ELISA assays also confirmed the robust upregulation of circulating sENG in the AAV6-mENG group relative to that in the control group (Fig. S1D). These serum sENG concentrations were consistent with those previously reported in other established preeclampsia mouse models [25, 27]. Phenotypically, AAV6-mENG mice developed the key hallmarks of PE, including a progressive and sustained elevation in MAP (Fig. 1A) and marked 24-hour proteinuria (Fig. 1B). Additionally, sENG overexpression resulted in compromised reproductive outcomes, characterized by reduced litter size, mean fetal weight and placental weight per litter (Fig. 1D-F). Then we analyzed high-quality scRNA-seq data from control and PE placental samples following rigorous quality control (Fig. S2) and then identified 12 distinct cell types (Fig. S3), GO/KEGG enrichment analysis demonstrated that the identified differentially expressed genes (DEGs) were significantly enriched in apoptosis (Bcl-2 family protein complex, apoptotic signaling; Fig. S4A). Given the well-established role of dysregulated apoptosis in placental dysfunction, coupled with our bioinformatic analyses, we performed TUNEL and IHC staining on mouse placentas from both groups. Quantitative TUNEL analysis confirmed markedly increased apoptotic cell abundance, mainly localized to the placental labyrinth region in the AAV6-mENG group (Fig. 1F, G). IHC staining for BAX, cleaved caspase-3, and Bcl-2 further validated this pro-apoptotic shift (Fig. 1H), pro-apoptotic BAX and cleaved caspase-3 were upregulated (Fig. 1I, J), while anti-apoptotic Bcl-2 was downregulated in the labyrinth zone of experimental placentas (Fig. 1K). To characterize cellular heterogeneity, violin plots were used to compare pathway scores for “apoptosis-induced DNA fragmentation” across cell types between control and PE groups, revealing a substantial increase specifically in trophoblasts(Fig. S4B). Collectively, these findings suggest that increased sENG levels contribute to the progression of PE and are associated with robust placental apoptosis, primarily observed within trophoblast populations.

### sENG exerts dose-dependent cytotoxicity and functional impairment in human trophoblasts

**Fig. 2:**
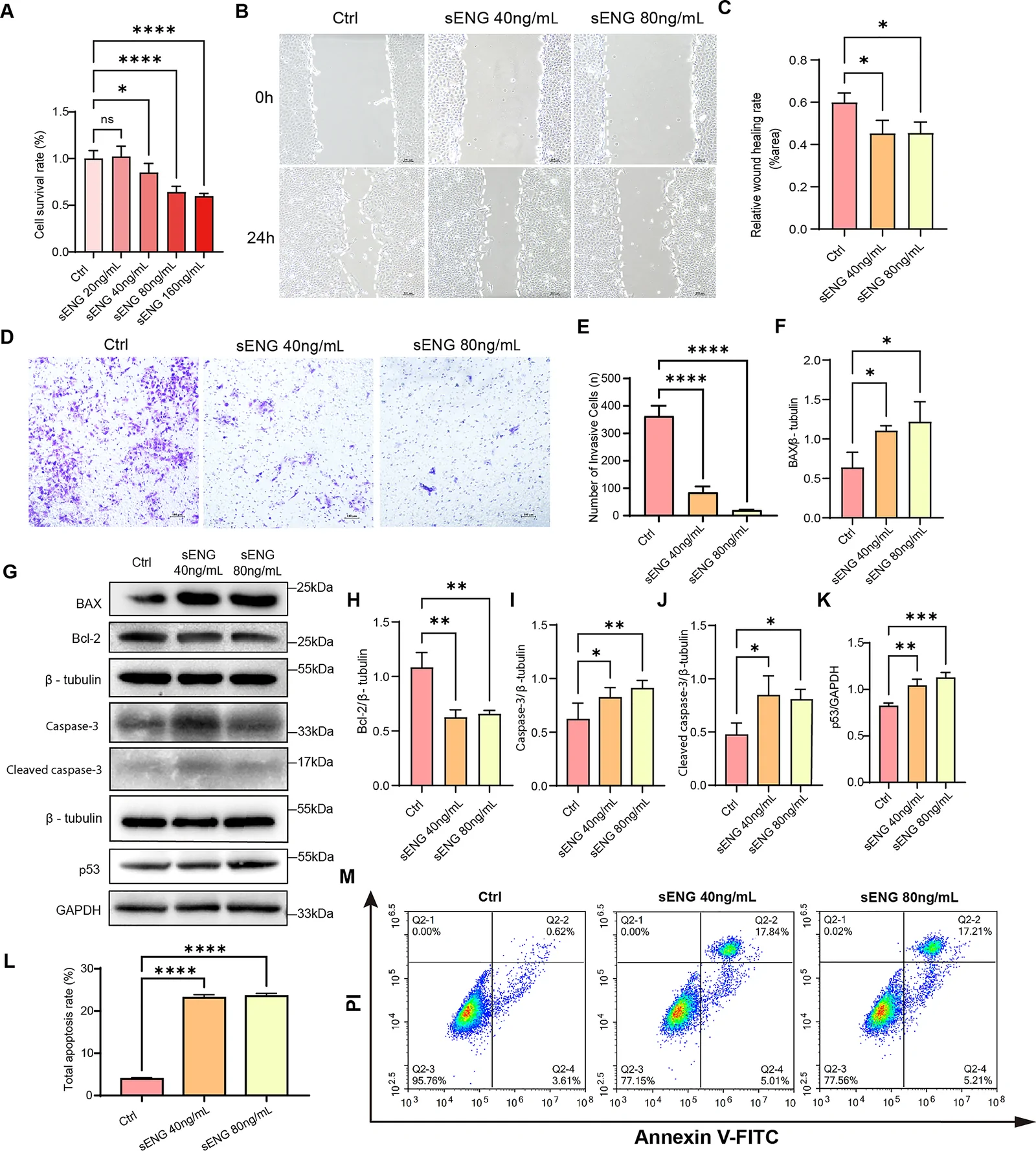
sENG inhibits proliferation, migration, invasion, and induces apoptosis in HTR-8/SVneo cells. (**A**) Cell viability of HTR-8/SVneo after 0-160 ng/mL sENG treatment by CCK-8 assay; (**B**,**C**) Wound-healing assay and quantification of wound healing rate of HTR-8/SVneo stimulated by sENG; (**D**,**E**) Transwell invasion assay and counting of invasive HTR-8/SVneo cells with sENG stimulation; (**F**-**K**) Western blot and quantification of apoptosis-related proteins (BAX, Bcl-2, caspase-3, cleaved caspase-3, p53), β-tubulin or GAPDH served as loading controls. (**L**,**M**) Representative flow cytometry dot plots of Annexin V-FITC/PI double staining and quantitative statistical analysis of the total apoptotic rate in HTR-8/SVneo stimulated by sENG. The significance of all data was determined using one-way ANOVA followed by Dunnett’s post-hoc test. Data were presented as the mean ± SEM of three independent experiments. *P < 0.05, ** *P* < 0.01, ***P < 0.001, ****P < 0.0001

To isolate the direct effects of sENG from the complex maternal-fetal microenvironment, we treated the human trophoblast cell line HTR-8/SVneo with recombinant sENG. As shown in Fig. 2A, sENG reduced cell viability in a dose-dependent manner, achieving significance at concentrations of ≥ 40 ng/mL. Consequently, 40 ng/mL (*P* = 0.0146) and 80 ng/mL (*P* < 0.0001) were selected for subsequent assays. Functional evaluation using wound-healing and Transwell assays demonstrated that sENG treatment significantly compromised trophoblast migration and invasion, as reflected by a diminished wound closure rate (Fig. 2B, C) and a reduced number of invasive cells (Fig. 2D, E). Mechanistically, Western blot analysis confirmed the induction of a pro-apoptotic phenotype: sENG treatment upregulated the pro-apoptotic markers BAX, caspase-3, cleaved caspase-3 and p53, while concurrently suppressing the anti-apoptotic protein Bcl-2 (Fig. 2F-K). Annexin V-FITC/PI dual staining flow cytometry was performed to quantify total apoptosis in Ctrl, 40 ng/mL sENG and 80 ng/mL sENG groups. The results revealed that treatment with 40 ng/mL or 80 ng/mL sENG markedly elevated the total apoptotic ratio of HTR-8/SVneo cells, mainly driven by a sharp increase in early apoptotic cell population, compared with the control group (Fig. 2L, M). Consistent with the impaired placental phenotype in AAV6-mENG mice, these data demonstrate that sENG induces apoptosis and suppresses the functional capacity of trophoblasts.

### sENG overexpression disrupts the CD39-ATP axis in the mouse placenta

We next interrogated the molecular mechanisms linking sENG to trophoblast apoptosis. According to GO/KEGG enrichment analysis of PE patients and controls(Fig. S4A), TGF-β signaling (cellular response to TGF-β stimulus, response to TGF-β), purine metabolism and cellular stress responses were also enriched. Fig. S4C showed a marked reduction in “ATP Biosynthetic Process” scores in trophoblasts of PE specimens. Given the pivotal role of Smad2/3 in TGF-β signaling, while ENTPD1 (CD39) is the primary representative gene enriched in purine metabolism and cellular stress response pathways, we mapped their cell-type-specific expression profiles. The results indicated a potential functional convergence of these molecules in PE pathogenesis (Fig. S4D-E).

**Fig. 3:**
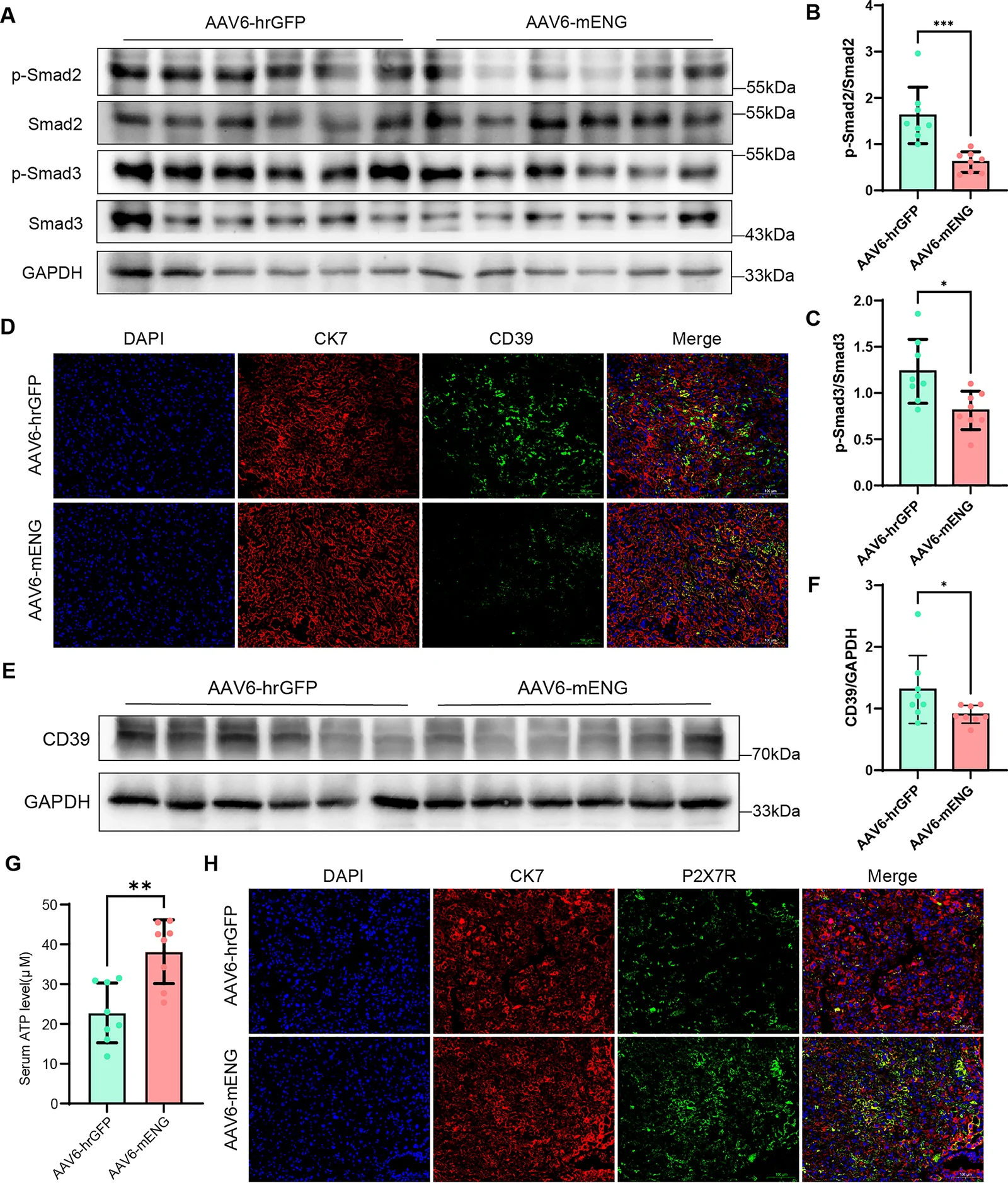
Overexpression of sENG in mouse placenta downregulates p-Smad2/3 and CD39 expression, induces eATP accumulation and P2 × 7R activation. AAV6-hrGFP: Pregnant mice were injected with AAV6-hrGFP as the control group (*n* = 8). AAV6-mENG: Pregnant mice were injected with an equivalent volume of AAV6-mENG as the experimental group (*n* = 8). (**A**-**C**) Representative Western blot images and quantitative analysis of p-Smad2, Smad2, p-Smad3, and Smad3 protein expression in placental tissue at D19; GAPDH was used as the loading control (two placentas per dam were randomly collected, with mean values used for statistical comparison); (**D**) IF staining of DAPI (blue, nuclei), CK7 (red, trophoblast marker), and CD39 (green), merged images show co-localization signals (scale bar:100 μm); (**E**,**F**) Representative Western blot images and quantitative analysis of CD39 protein expression in placental tissue at D19; GAPDH was used as the loading control (two placentas per dam were randomly collected, with mean values used for statistical comparison); (**G**) Serum eATP levels in maternal blood at D19; (**H**) IF staining of DAPI (blue, nuclei), CK7 (red, trophoblast marker), and P2 × 7R (green), merged images show co-localization signals (scale bar:100 μm); The significance of all data was determined using Student’s t (B, G) or non-parametric tests(C, F). Data were presented as the mean ± SEM. *P < 0.05, ** *P* < 0.01, ****P < 0.0001

Subsequently, we detected the expression of the aforementioned key molecules in placentas of the mouse model. Western blot analysis showed significant suppression of phosphorylated Smad2/3 (p-Smad2/3) in the placentas of AAV6-mENG mice compared to AAV6-hrGFP controls (Fig. 3A–C). IF staining localized CD39 expression specifically to cytokeratin 7 (CK7)-positive trophoblasts. Consistently, both IF and Western blot analyses demonstrated a significant reduction in CD39 protein levels in the AAV6-mENG group (Fig. 3D–F). As shown in Fig. 3G, serum ATP levels were markedly elevated in the AAV6-mENG group compared to the control group. Furthermore, we examined the expression of the P2X7R, a key mediator of ATP-induced cell death. IHC analysis revealed significantly elevated P2X7R levels in the AAV6-mENG placentas (Fig. 3H). These in vivo data suggest that sENG overexpression impairs the Smad2/3-CD39 signaling axis, leading to a molecular environment that favors eATP accumulation and subsequent P2X7R activation.

### sENG promotes eATP accumulation by suppressing the TGF-β-Smad2/3-CD39 pathway

**Fig. 4:**
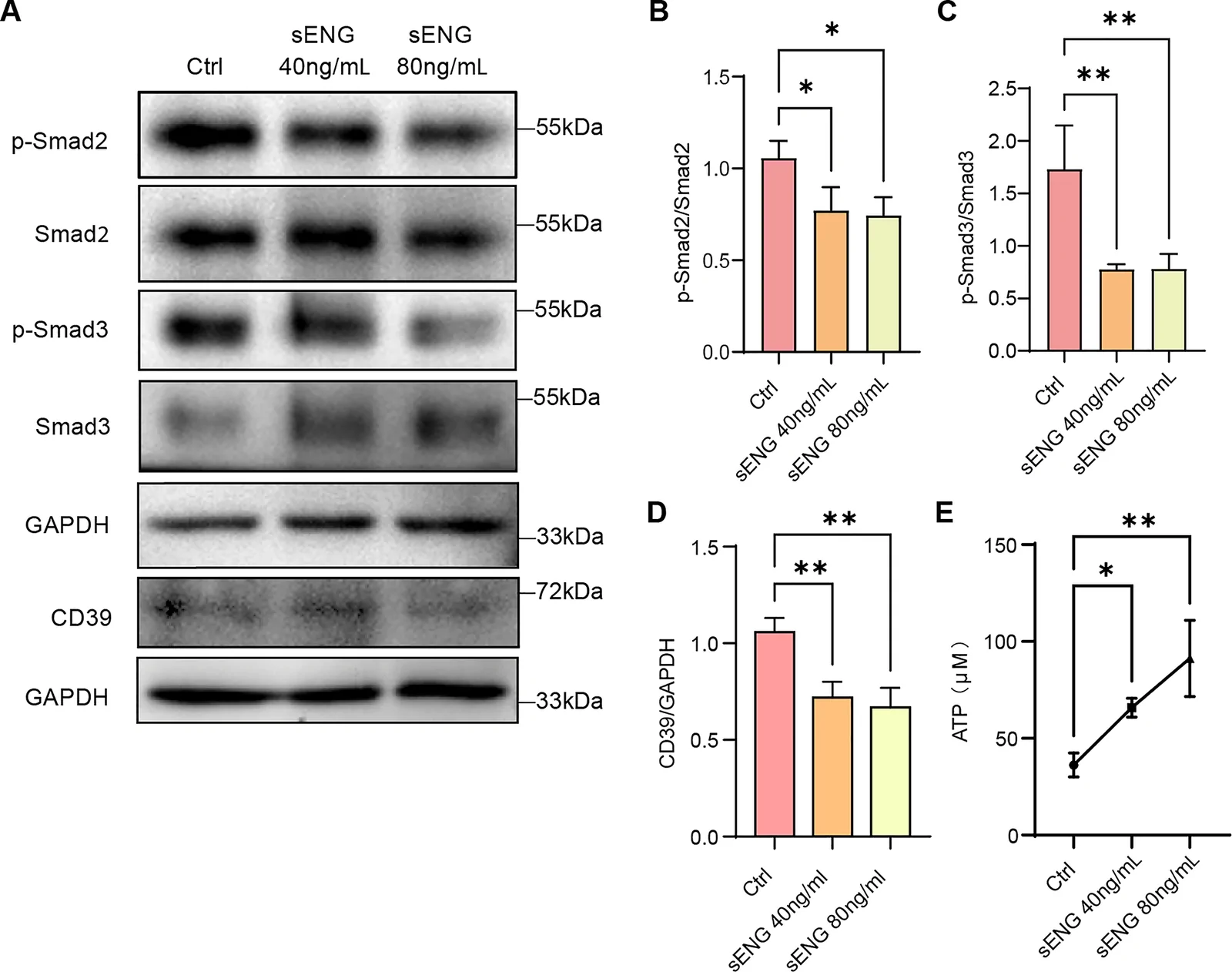
Changes in the TGF-β-Smad2/3 pathway, CD39 expression and eATP levels in HTR-8/SVneo cells following treatment with 0–80 ng/mL sENG (*n* = 3). (**A**-**D**) The expression levels of p-Smad2/Smad2, p-Smad3/Smad3 and CD39 were detected by western blotting in each group; (**E**) eATP levels in the cell supernatants of HTR-8/SVneo in each group. The significance of all data was determined using one-way ANOVA, followed by Dunnett’s post-hoc test.Data were presented as the mean ± SEM of three independent experiments. *P < 0.05, ** *P* < 0.01

Based on scRNA-seq data identifying ATP metabolism as the top dysregulated pathway and the observed CD39 downregulation in both PE patients and AAV6-mENG mice (Fig. S4, Fig. 3), we hypothesized that sENG mediates eATP accumulation via suppression of the TGF-β-Smad2/3-CD39 axis. We first validated the regulation of eATP by CD39 using the inhibitor ARL67156 (Fig. S5). In HTR-8 cells, sENG treatment significantly reduced the phosphorylation of both Smad2 (Fig. 4A, B) and Smad3 (Fig. 4A, C), and dose-dependently downregulated CD39 protein expression (Fig. 4A, D). Crucially, this molecular suppression resulted in functional consequences, as sENG treatment induced a significant accumulation of eATP in the culture supernatants (Fig. 4E; *P* = 0.0443, *P* = 0.0027), with the 80 ng/mL concentration driving a nearly two-fold increase relative to the controls. These data demonstrate that sENG impairs eATP clearance in the trophoblast microenvironment by inhibiting the TGF-β-Smad2/3-dependent CD39 expression.

### TGF-β1 restores the Smad2/3-CD39 axis and rescues sENG-induced trophoblast dysfunction

**Fig. 5:**
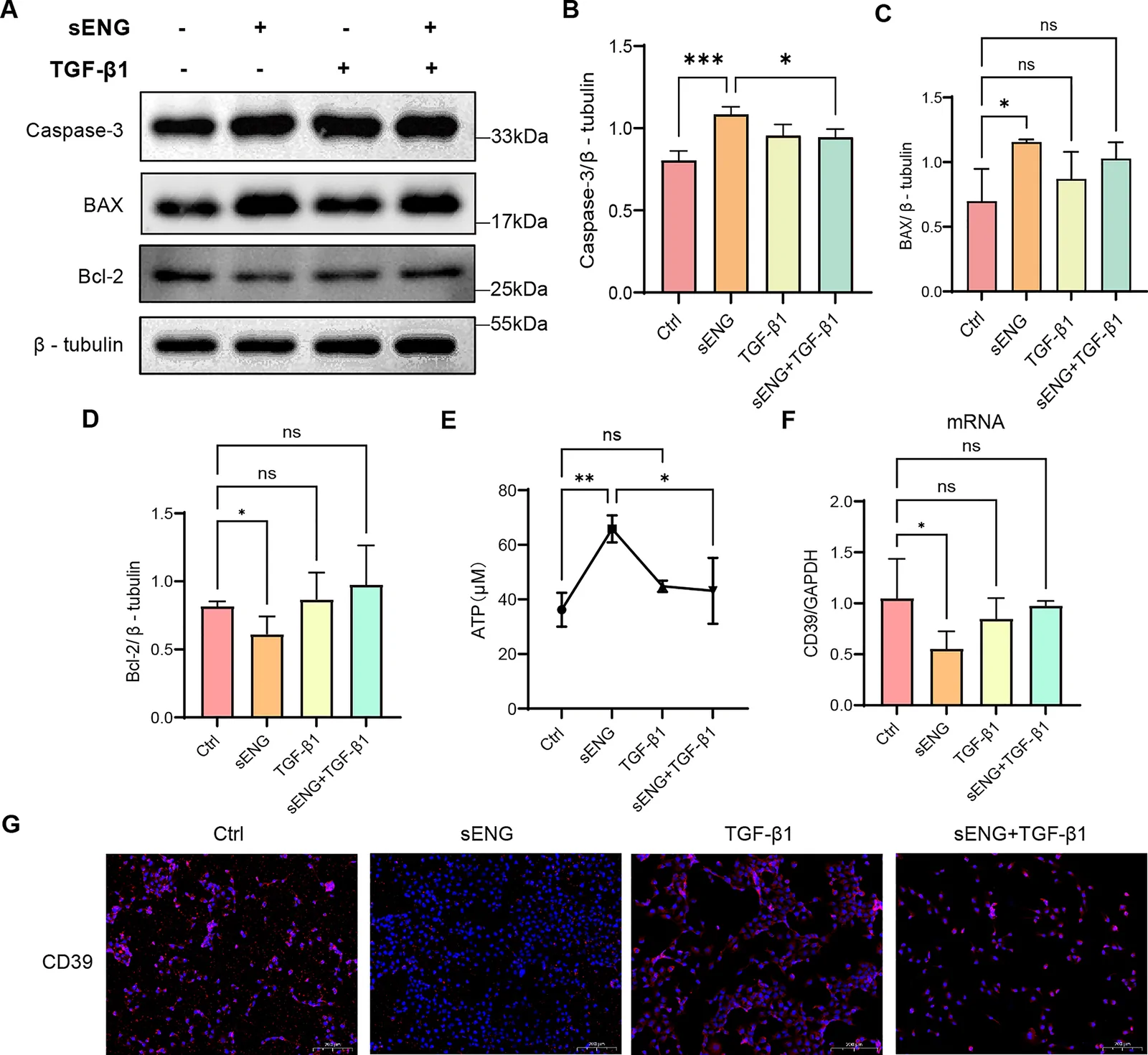
TGF-β1 abrogated the effects of sENG on the Smad2/3-CD39 axis and eATP levels in HTR-8/SVneo. Ctrl: Cells were cultured in basic medium only; sENG: Cells were treated with 40ng/mL sENG in basic medium; TGF-β1: Cells were treated with 5ng/mL TGF-β1 alone in basic medium; sENG + TGF-β1: Cells were co-treated with sENG and TGF-β1 in basic medium. (**A**-**D**) Western blot analysis of Caspase-3, BAX and Bcl2 in each group, with β-tubulin as loading control; (**E**) eATP level in the cell supernatants of HTR-8/SVneo in each group; (**F**) qPCR analysis of CD39/GAPDH level in each group; (**G**) IF staining of CD39 (red) in four groups, nuclei were counterstained with DAPI (blue), scale bar:200 μm. The significance of all data was determined using one-way ANOVA followed by Tukey’s post-hoc test. Data were presented as the mean ± SEM of three independent experiments. *P < 0.05, ** *P* < 0.01,***P < 0.001

To substantiate the causal role of the TGF-β-Smad2/3 pathway in sENG-mediated pathology, we performed rescue experiments using exogenous TGF-β1. Given the comparable effects observed at both sENG concentrations, 40 ng/mL was utilized for these assays. While sENG treatment alone elicited a robust pro-apoptotic response—characterized by Caspase-3 (Fig. 5A, B) and BAX (Fig. 5A, C) upregulation and Bcl-2 downregulation (Fig. 5A, D), co-administration of TGF-β1 effectively abrogated these changes, restoring apoptotic regulators to near-baseline levels. Functionally, TGF-β1 co-treatment significantly reversed sENG-induced eATP accumulation (Fig. 5E, *P* = 0.0155). Mechanistically, this rescue effect was underpinned by the restoration of CD39 expression at both the mRNA (Fig. 5F) and protein (Fig. 5G) levels. These findings provide supportive evidence that reactivating the TGF-β-Smad2/3 pathway is sufficient to reverse sENG-induced CD39 downregulation, prevent eATP accumulation, and protect trophoblasts from apoptosis.

### CD39 overexpression rescues sENG-induced apoptosis and ATP accumulation

**Fig. 6:**
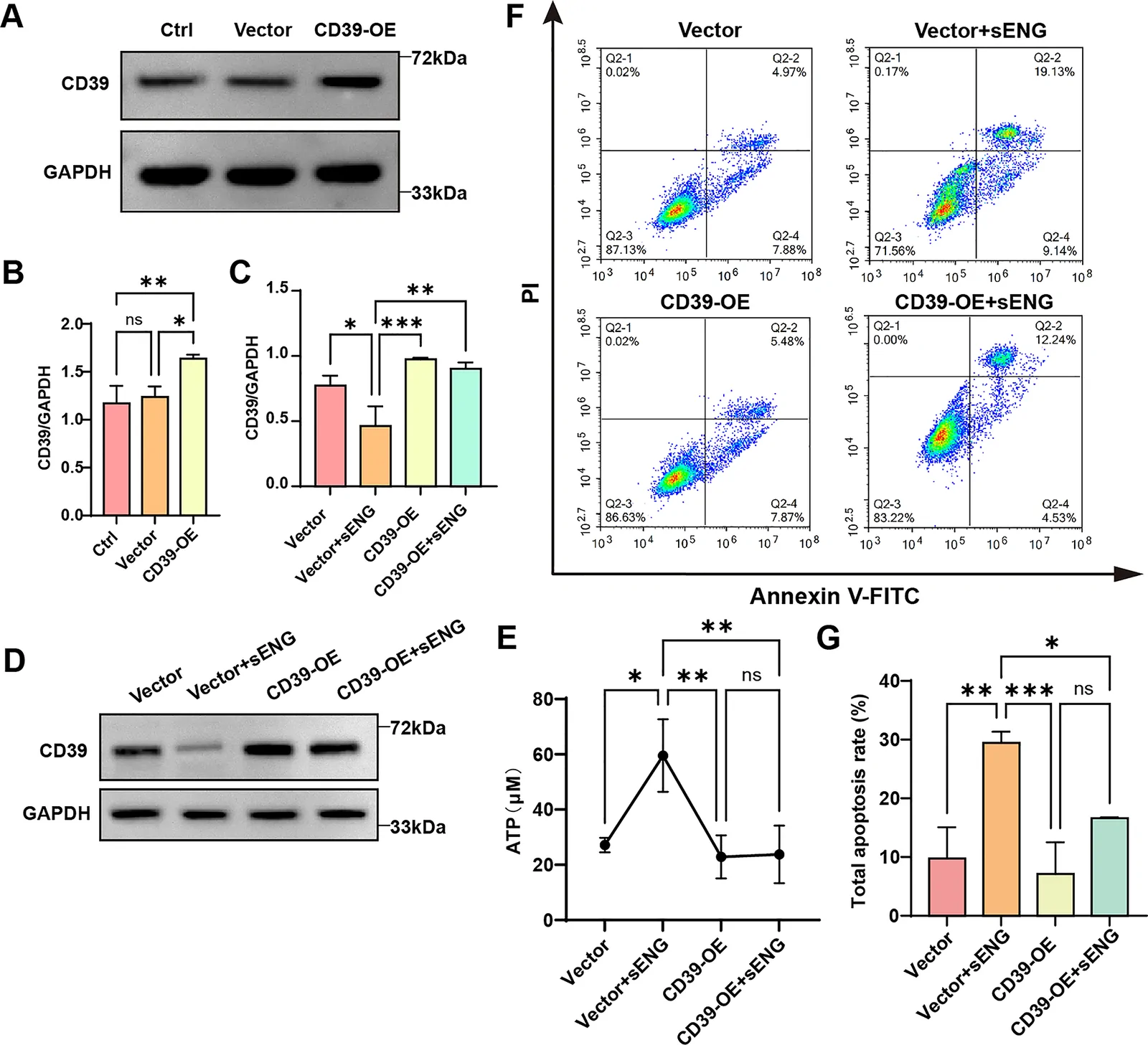
Effects of sENG on cell apoptosis and extracellular ATP levels in CD39-overexpressing HTR-8/SVneo cells. Ctrl: wild-type HTR-8/SVneo cells; Vector: HTR-8/SVneo cells transfected with empty overexpression vector; Vector+sENG: empty vector-transfected cells treated with sENG; CD39-OE: HTR-8/SVneo cells stably overexpressing CD39; CD39-OE+sENG: CD39-overexpressing cells treated with sENG. (**A**, **B**) Representative western blots and quantification of CD39 expression in Ctrl, Vector and CD39-OE groups; (**C**, **D**) Representative western blot images and quantification of CD39 in Vector and CD39-OE cells upon sENG stimulation; (**E**) Extracellular ATP levels in cell culture supernatant of each group; (**F**, **G**) Annexin V-FITC/PI flow cytometry dot plots and statistical quantification of total cell apoptotic rate across all groups. The significance of all data was determined using one-way ANOVA followed by Tukey’s post-hoc test. Data were presented as the mean ± SEM of three independent experiments. *P < 0.05, ** *P* < 0.01,***P < 0.001

To investigate the function of CD39 in purine metabolism, we generated CD39-overexpressing (CD39-OE) HTR8 trophoblast cells. Western blot verified markedly higher CD39 protein abundance in CD39-OE cells versus Ctrl and Vector groups (Fig. 6A-B). We further treated Vector and CD39-OE cells with sENG. sENG stimulation markedly downregulated endogenous CD39 in Vector cells, consistent with our observations in wild-type HTR8 cells, while CD39 protein levels remained stable in the CD39-OE group (Fig. 6C-D). eATP measurement showed sENG strongly elevated eATP in Vector cells, whereas CD39 overexpression markedly reduced this eATP accumulation (Fig. 6E). Annexin V/PI flow cytometry assessed apoptosis: sENG induced severe trophoblast apoptosis in Vector cells, while CD39 overexpression significantly reversed sENG-triggered cell death (Fig. 6F-G). In summary, CD39 eliminates sENG-induced eATP accumulation and protects trophoblasts against sENG-mediated apoptosis.

### Extracellular ATP mediates sENG-induced toxicity via P2X7 receptor activation

**Fig. 7:**
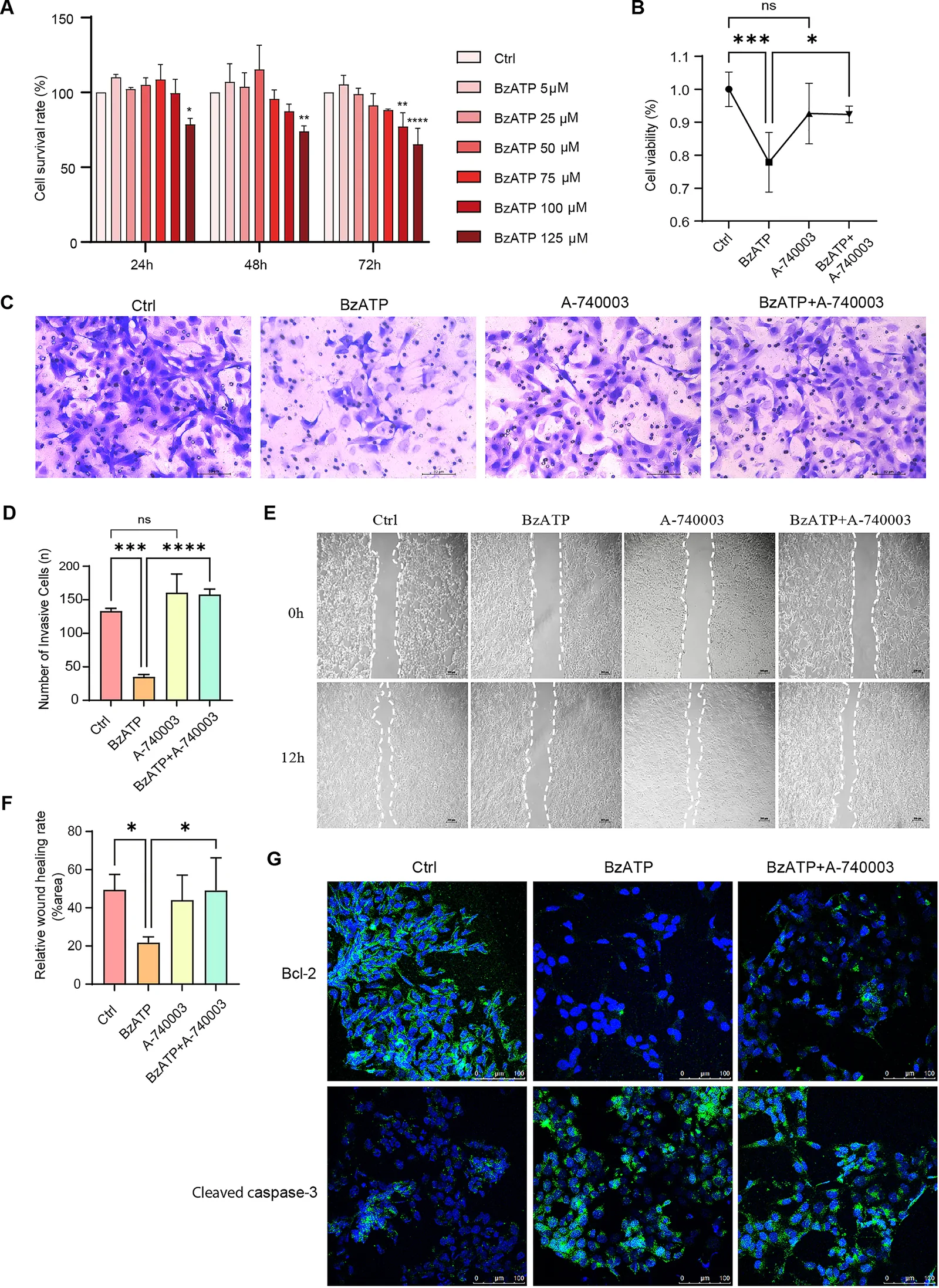
Function changes and apoptosis levels of HTR-8/SVneo stimulated by BzATP with or without A-740,003. Ctrl: Cells were cultured in basic medium only; BzATP: Cells were treated with 125µM BzATP in basic medium; A-740,003: Cells were treated with 10µM A-740,003 alone in basic medium; BzATP + A-740,003: Cells were co-treated with BzATP and A-740,003 in basic medium. (**A**) Cell viability of HTR-8/SVneo after treatment with various concentrations of BzATP by CCK-8 assay(24 h/48 h/72 h); (**B**) Cell viability quantification in each group; (**C**-**D**) Transwell invasion assay and counting of invasive HTR-8/SVneo in each group; (**E**-**F**) Wound-healing assay and quantification of wound healing rate of HTR-8/SVneo in each group; (**G**) IF staining of Bcl-2 and cleaved caspase-3 in each groups, scale bar:100 μm. The significance of all data was determined using one-way ANOVA followed by Tukey’s post-hoc test. Data were presented as the mean ± SEM of three independent experiments.*P < 0.05, ***P < 0.001,****P < 0.0001

We next investigated whether P2X7R acts as the downstream effector of sENG-induced eATP accumulation. We employed the selective P2X7R agonist BzATP and the antagonist A-740,003 for mechanistic dissection. CCK8 assays showed that BzATP (5–125 µM) triggered a dose- and time-dependent reduction in HTR-8 viability, with 125 µM identified as the effective dose (Fig. 7A). Importantly, co-treatment with A-740,003 reversed this BzATP-induced cytotoxicity (Fig. 7B). Furthermore, BzATP stimulation significantly suppressed trophoblast invasion (Fig. 7C, D) and migration (Fig. 7E, F); these functional deficits were rescued by A-740,003 co-treatment, confirming the specific involvement of P2 × 7R. At the molecular level, IF analysis revealed that BzATP treatment downregulated Bcl-2 and upregulated cleaved caspase-3 (Fig. 7G), a profile corrected by A-740,003. Consistent trends were observed in cleaved caspase-3 activity assays and Ki67 staining (Figure S6). Collectively, these data establish that eATP promotes trophoblast apoptosis and functional impairment specifically through P2X7R activation.

### Clinical profiling of the sENG-TGF-β-CD39-P2X7R axis in preeclamptic placentas

**Fig. 8:**
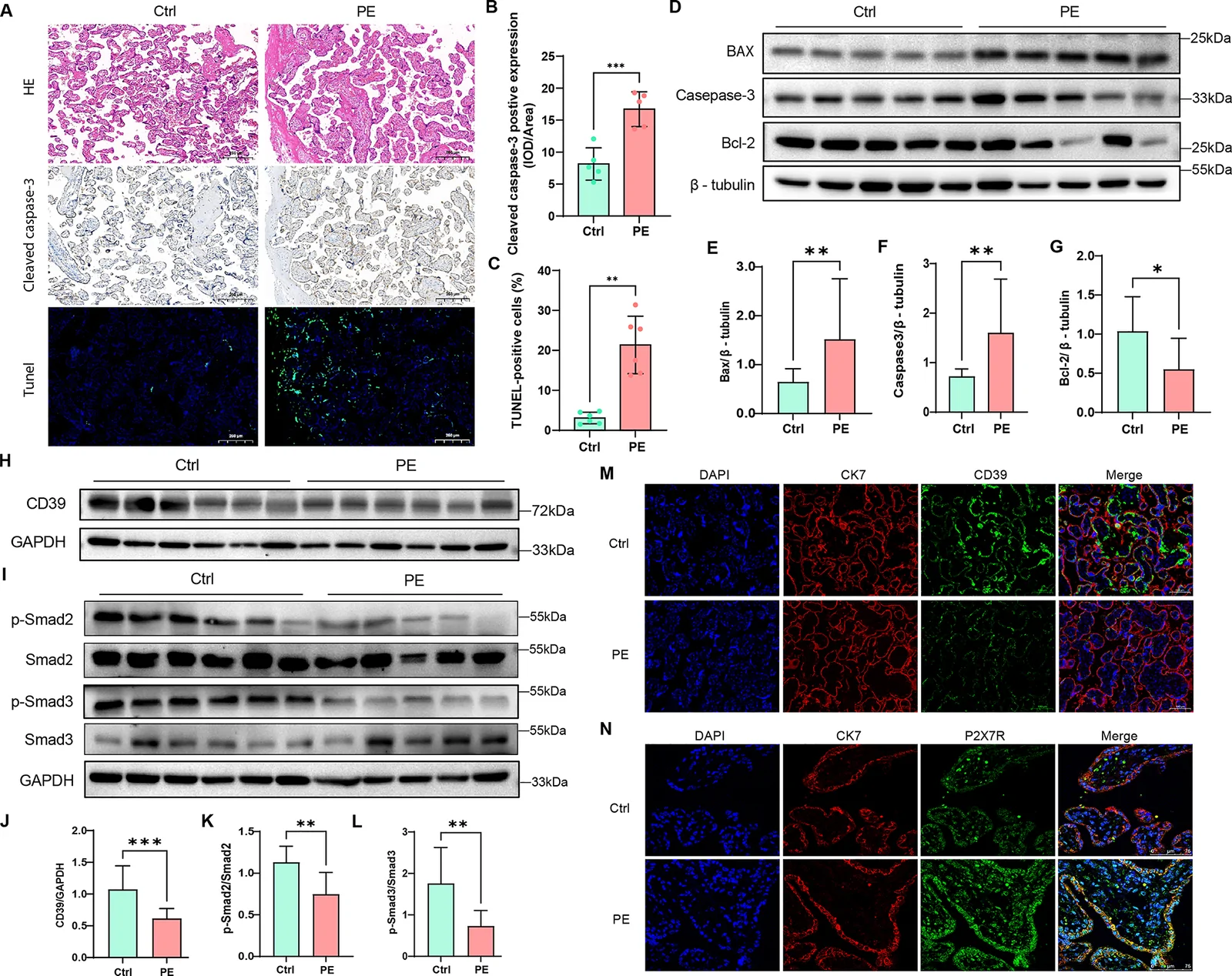
P2X7R-mediated apoptosis, CD39 expression, and Smad2/3 signaling in placenta tissues from controls and PE patients. Ctrl: Placenta tissues from 10 healthy pregnant women; PE: Placenta tissues from 10 patients with PE. (**A**-**C**) Representative HE, IHC of cleaved caspase-3 and TUNEL staining images and analysis of placentas from two groups, scale bar:250 μm); (**D**-**G**) Western blot analysis of BAX, cleaved caspase-3, and Bcl-2 protein levels, with β-tubulin as a loading control (*n* = 10); (**H**-**L**)Western blot analysis of CD39 and p-Smad2/3 expression, normalized to GAPDH (*n* = 10); (**M**) IF staining of DAPI (blue, nuclei), CK7 (red, trophoblast marker), and CD39 (green) in placenta, merged images indicate co-localization; scale bar = 100 μm. (**N**) IF staining of DAPI (blue, nuclei), CK7 (red, trophoblast marker), and P2X7R (green), merged images show co-localization signals; scale bar = 75 μm. The significance of all data was determined using Student’s t-(**B**,**G**,**K**,**L**) or non-parametric tests (**C**-**F**,**J**). Data were presented as the mean ± SEM. *P < 0.05, ** *P* < 0.01,***P < 0.001

To further explore the clinical relevance of our experimental observations, we profiled placental tissues from PE patients and matched normotensive controls. The baseline characteristics of healthy controls (*n* = 10) and PE patients (*n* = 10) showed no significant differences in maternal age, BMI, parity, or gestational age at delivery (all *P* > 0.05). As expected, PE patients exhibited significantly higher pre-delivery systolic and diastolic blood pressure (*P* < 0.05), while healthy control subjects did not undergo 24-hour urinary protein quantitation due to the absence of comorbidities. Placentas from PE patients exhibited a distinct pro-apoptotic molecular signature, characterized by the IHC of cleaved caspase-3 and TUNEL staining (Fig. 8A-C). WB also revealed a significant upregulation of BAX (Fig. 8D, E, *P* = 0.0062) and caspase-3 (Fig. 8D, F, *P* = 0.003), and downregulation of Bcl-2 (Fig. 8D, G, *P* = 0.0178). Corroborating our preclinical models, CD39 protein expression and p-Smad2/3 levels were profoundly reduced in PE tissues (Fig. 8H-L). IF analysis confirmed the co-localization of CD39 with CK7-positive trophoblasts and verified its reduced abundance in PE samples (Fig. 8M). Finally, consistent with the consequences of CD39 deficiency and eATP accumulation, IF revealed a marked upregulation of P2X7R in PE tissues (Fig. 8N). These results demonstrate that the sENG-TGF-β-Smad2/3-CD39-eATP-P2X7R axis is dysregulated in human PE, supporting the clinical relevance of this signaling cascade.

## Discussion

PE, a pregnancy-specific hypertensive disorder, remains a leading cause of maternal and perinatal morbidity and mortality worldwide [28]; however, its pathogenic mechanisms have not been fully elucidated [29]. sENG, a soluble isoform of ENG that acts as a co-receptor for the TGF-β superfamily, has long been recognized as a key anti-angiogenic factor in the pathogenesis of PE [30]. According to Leaños-Miranda et al. [20], circulating concentrations of sENG could be a marker for assessing the severity of PE and are associated with an increased risk of adverse outcomes. Moreover, transgenic mice overexpressing human sENG showed a PE-like phenotype, such as increased levels of MAP and renal damage [31], indicating that sENG not only exerts an endothelial injury effect after placenta ischemia but also has a direct association with the occurrence of PE. In the present study, we integrated in vivo mouse models, in vitro trophoblast cell experiments, and human placental tissue analyses to propose a previously unrecognized identify a novel sENG-TGF-β-Smad2/3-CD39-eATP-P2X7R signaling axis that may contribute to trophoblast apoptosis and functional impairment in PE. The placenta mediates maternal-fetal nutrient exchange and immune tolerance, which critically depend on the normal physiological activity of trophoblast cells [32]. Meanwhile, it has been widely proven that insufficient invasion of extravillous trophoblasts, together with syncytiotrophoblast stress, is a key mediator of the onset and progression of PE [33, 34]. Our bioinformatics analysis indicated enrichment of apoptosis-related signatures in placental tissue was markedly elevated in preeclamptic patients relative to normal pregnant controls. Given the established association between sENG and PE, we hypothesized that sENG may impair trophoblast function and thereby compromise placental homeostasis. However, the direct role of sENG in regulating trophoblast biology remains insufficiently characterized. Our in vivo data demonstrated that sENG overexpression in mice induced typical PE phenotypes accompanied by significant trophoblast apoptosis. Our in vitro results showed that sENG induced apoptosis in a dose-dependent manner and impaired proliferation, migration, and invasion, with an onset dose of 40 ng/mL in HTR-8/SVneo cells. Notably, the functional concentration of sENG used in our in vitro assays was consistent with the circulating levels detected in patients with PE, according to a study published in the New England Journal of Medicine [35]: the mean serum levels in women with preterm PE and PE at term were 46.4 ng/mL and 31.0 ng/mL, respectively, compared with 9.8 ng/mL and 13.3 ng/mL in controls. These findings support the possibility that sENG may function as an active contributor to placental injury rather than merely reflecting secondary placental damage in PE. Similarly, Pérez-Roque et al. [25] found that wild-type pregnant mice carrying human sENG-expressing transgenic fetuses (fWT(hsENG+)) presented high plasma levels of sENG and placental alterations, and the presence of sENG in vitro reduced both proliferation and invasiveness of the trophoblast cell line JAR. However, they did not find evidence of a relationship between sENG and trophoblast apoptosis, which appears to differ from our observations. Given that the JAR cell line is a tumor cell line, the apoptosis levels observed in this cell model may not fully reflect the physiological characteristics of normal trophoblast cells.

TGF-β signaling is a well-characterized regulator of cellular behavior, with the Smad2/3 pathway serving as the canonical downstream effector of this signaling pathway. It plays a critical role in placental development and is involved in many diseases, such as PE [36]. ENG, the membrane-bound precursor of sENG, is a cell surface co-receptor of TGF-β signaling, and sENG acts as a decoy receptor to sequester TGF-β ligands such as TGF-β1, thereby inhibiting canonical TGF-β-Smad2/3 signaling in target cells [37, 38]. Although the interaction between sENG and TGF-β-Smad2/3 has been extensively documented, the regulation of the TGF-β-Smad2/3 pathway and its molecular effectors of this pathway that mediate trophoblast dysfunction in PE remain unclear. In the present study, we identified alterations in the TGF‑β signaling pathway and differential expression of Smad2/3 and CD39 in trophoblasts from PE patients and controls through bioinformatics analysis. Interestingly, CD39 downregulation was also observed in Hofbauer cells and stromal cells by immunofluorescence staining of placental tissues, which contributes to the accumulation of eATP in the microenvironment of trophoblasts, may involve responses of multiple placental cell types to elevated sENG. CD39 is a key regulator of purinergic signaling that has been implicated in various pregnancy complications, such as recurrent pregnancy loss [18, 39]. Its role in PE has been largely unexplored, with only a small number of studies reporting that the expression of CD39 is decreased in PE cases compared to healthy controls at the mRNA and protein levels [40]. Our findings align with these preliminary clinical observations and extend these observations by providing mechanistic evidence for CD39 downregulation in PE. Specifically, sENG suppressed the TGF-β-Smad2/3 pathway, and exogenous sENG-mediated apoptosis and CD39 downregulation were rescued by TGF-β1. According to previous studies, the expression and activity of CD39 are tightly controlled by transcriptional and post-transcriptional mechanisms in various cell types [41, 42]. For example, TGF-β increases CD39 expression on Tregs via the activation of TGFBRII/TGFBRI, Smad2 and the transcription factor CREB [24]; CD39-specific antisense regulates CD39 by interacting with nucleolin and heterogeneous-nuclear-ribonucleoprotein-A1 in Treg and Th17-cells of patients with Crohn’s disease [43]. To our knowledge, the present study is the first to establish a regulatory link between TGF-β–Smad2/3 signaling and CD39 expression within placental trophoblasts, and that sENG-mediated inhibition of this pathway leads to significant CD39 downregulation. Nevertheless, the precise transcriptional mechanisms by which TGF-β/Smad2/3 signaling regulates CD39 expression require further investigation.

CD39, an ectonucleotidase responsible for eATP hydrolysis, is downregulated and associated with increased eATP accumulation in the extracellular microenvironment of various cell types [44, 45]. Consistent with this well-established relationship, our experiments verified the ATP-modulating function of CD39 in trophoblasts and further revealed that sENG-induced CD39 downregulation in trophoblasts led to a marked increase in eATP levels both in vitro and in vivo.CD39 overexpression rescue assays further support the involvement of CD39 loss in sENG-induced eATP accumulation and trophoblast apoptosis, as restoring CD39 reduced ATP accumulation and partially alleviated cell death. Although CD39 overexpression could not be achieved in our preeclampsia mouse model, prior work has reported that CD39-knockout pregnant mice exhibit drastically elevated risk of fetal loss. This observation aligns with our findings and collectively supports the protective role of CD39 during gestation [18]. eATP exerts its biological effects by binding to and activating plasma membrane purinergic receptors, which are divided into two major families: ionotropic P2X receptors (P2X1-P2X7) and metabotropic P2Y receptors (P2Y1, P2Y2, P2Y4, P2Y6, P2Y11-P2Y14) [46] that also exhibit distinct tissue expression patterns and functional roles. According to a previous study, only a subset of receptors, including P2X7R, is expressed in the human placenta. Notably, P2X7R is crucial in inflammation, cell death, and immune responses, all of which have been confirmed to participate in the pathogenesis of PE [47]. However, the expression and underlying mechanisms of P2X7R in trophoblast cells in PE remain largely unexplored. Our functional experiments, including P2X7R inhibition, showed that eATP-mediated trophoblast apoptosis and functional impairment were markedly abrogated by targeting P2X7R, demonstrating its vital role in sENG-induced trophoblast dysfunction. These findings are consistent with those of previous studies: CD39 expression in macrophages limits P2X7R-mediated pro-inflammatory responses by scavenging eATP and ultimately generating adenosine [48].

Ultimately, our identification of the sENG–eATP–P2X7R signaling axis offers a novel mechanistic perspective to bridge localized placental injury and the systemic immune-inflammatory cascade characteristic of PE. Accumulating evidence suggests that excess sENG disrupts angiogenic homeostasis (e.g., via neutralizing BMP9) and drives nuclear factor κ-light-chain-enhancer of activated B cells (NF-κB)-mediated inflammatory cascades, triggering persistent endothelial injury and oxidative stress [29, 37, 49–51]. Importantly, our study revealed a synergistic mechanism: the excessive accumulation of eATP, combined with abundant intracellular factors released by apoptotic trophoblasts, forms potent DAMPs. Upon activating receptors like P2X7R, these DAMPs not only exacerbate localized placental apoptosis but also provoke broad inflammatory responses. Substantial pro-inflammatory cytokines liberated during these processes likely act synergistically with sENG’s direct signaling to further compromise systemic endothelial homeostasis and exacerbate cellular damage [52–55]. In turn, the injured and ischemic placental tissue releases even more sENG into the maternal circulation [31, 56, 57]. This establishes a potent self-amplifying vicious cycle: sENG reduces CD39 and elevates eATP/DAMPs, aggravating inflammation, endothelial dysfunction and placental damage in a feedforward loop. Recent clinical studies have demonstrated that altered immune cell profiles represent reliable early biomarkers for PE [58], underscoring the necessity of elucidating immune activation mechanisms to identify candidate pathways for future mechanistic and translational studies. In line with these clinical findings, our mechanistic dissection of the intricate crosstalk between sENG-mediated trophoblast apoptosis and purinergic inflammatory signaling provides a plausible molecular basis to explain the early immune perturbations observed in PE patients. Collectively, this study deepens our fundamental understanding of PE pathogenesis and suggests that the CD39–ATP purinergic signaling cascade coupled with P2X7R may warrant further exploration as a clinically relevant pathway.

This study had some limitations that need to be addressed. Clinical validation was conducted in a relatively small, single-center cohort, and larger multicenter studies are needed to confirm the diagnostic and prognostic performance of the Smad2/3–CD39–P2X7R signature. The sample size of the single-cell RNA-seq analysis was also limited, and expanded sampling would yield more reliable biological inferences. Furthermore, although we believe that P2X7R is a key downstream effector in cases of elevated sENG, we did not directly test whether P2X7R blockade can therapeutically rescue placental/trophoblast injury in the setting of increased sENG in vivo. This issue will be specifically explored in future interventional studies.

## Conclusion

We propose an sENG–TGF-β–Smad2/3–CD39–eATP–P2X7R axis that contributes to trophoblast apoptosis and dysfunction in preeclampsia. Elevated sENG suppresses Smad2/3 signaling, downregulates CD39, increases extracellular ATP accumulation, and activates P2X7R-mediated trophoblast injury, as supported by evidence from in vivo, in vitro, and human placental analyses. These findings provide additional insight into the pathogenic mechanisms of PE and suggest that this axis deserves further investigation in future mechanistic and interventional studies.

## Supplementary Information

Below is the link to the electronic supplementary material.

Supplementary Material 1: Section 1. Baseline characteristics and related data; Sect. 2. Experimental reagents: antibodies and primers; Sect. 3. Supplementary experimental methods; Sect. 4. Supplementary results

Supplementary Material 2

## Data Availability

The datasets used and/or analysed during the current study are available from the corresponding author on reasonable request.

## Abbreviations

PE: Preeclampsia
sENG: Soluble Endoglin
TGF-β: Transforming growth factor-β
eATP: Extracellular ATP
AAV6: Adeno-associated virus serotype 6
P2X7R: P2X7 receptor
BMI: Body mass index
BMP: Bone morphogenetic protein
BzATP: 2’,3’-O-(4-Benzoylbenzoyl) adenosine 5’-triphosphate
CCK-8: Cell Counting Kit-8
CD39: Ectonucleoside triphosphate diphosphohydrolase 1 (ENTPD1)
DAB: 3,3’-Diaminobenzidine
DAMP: Damage-associated molecular pattern
DAPI: 4’,6-Diamidino-2-phenylindole
ECL: Enhanced chemiluminescence
ELISA: Enzyme-linked immunosorbent assay
ENTPD1: Ectonucleoside triphosphate diphosphohydrolase 1
FLT1: Fms-like tyrosine kinase 1
GO: Gene Ontology
HE: Hematoxylin and Eosin
TUNEL: Terminal Deoxynucleotidyl Transferase dUTP Nick End Labeling
IHC: Immunohistochemistry
IF: Immunofluorescence
KEGG: Kyoto Encyclopedia of Genes and Genomes
MAP: Mean arterial pressure
PBST: Phosphate-buffered saline with Tween-20
PBS: Phosphate buffer solution
qPCR: Quantitative real-time polymerase chain reaction
ROS: Reactive oxygen species
TNF-α: Tumor necrosis factor-alpha
CK7: Cytokeratin 7
DEGs: Differentially expressed genes
NF-κB: Nuclear factor κ-light-chain-enhancer of activated B cells

## References

[1] Rana S, Lemoine E, Granger JP, Karumanchi SA. Preeclampsia: Pathophysiology, Challenges, and Perspectives. Circ Res. 2019;124(7):1094–112. 10.1161/CIRCRESAHA.118.313276 30920918

[2] MacDonald TM, Walker SP, Hannan NJ, Tong S, Kaitu’u-Lino TJ. Clinical tools and biomarkers to predict preeclampsia. EBioMedicine. 2022;75:103780. 10.1016/j.ebiom.2021.103780 PMC8718967 34954654

[3] Dimitriadis E, Rolnik DL, Zhou W, Estrada-Gutierrez G, Koga K, Francisco RPV, et al. Pre-eclampsia. Nat Rev Dis Primers. 2023;9(1):8. 10.1038/s41572-023-00417-6 36797292

[4] Ives CW, Sinkey R, Rajapreyar I, Tita ATN, Oparil S. Preeclampsia-Pathophysiology and Clinical Presentations: JACC State-of-the-Art Review. J Am Coll Cardiol. 2020;76(14):1690–702. 10.1016/j.jacc.2020.08.014 33004135

[5] Jung E, Romero R, Yeo L, Gomez-Lopez N, Chaemsaithong P, Jaovisidha A, et al. The etiology of preeclampsia. Am J Obstet Gynecol. 2022;226(2s):S844–66. 10.1016/j.ajog.2021.11.1356 PMC8988238 35177222

[6] Xu X, Lai Y, Hua ZC. Apoptosis and apoptotic body: disease message and therapeutic target potentials. Biosci Rep. 2019;39(1). 10.1042/BSR20180992 PMC6340950 30530866

[7] Gong X, He W, Jin W, Ma H, Wang G, Li J, et al. Disruption of maternal vascular remodeling by a fetal endoretrovirus-derived gene in preeclampsia. Genome Biol. 2024;25(1):117. 10.1186/s13059-024-03265-z PMC11075363 38715110

[8] Liu L, Li X, Yang H, Xu F, Dong X. Bioinformatic Analysis of Apoptosis-Related Genes in Preeclampsia Using Public Transcriptomic and Single-Cell RNA Sequencing Datasets. J Inflamm Res. 2025;18:4785–812. 10.2147/JIR.S507660 PMC11992479 40224388

[9] Wang Y, Lv Q, Li J, Hu M, Li H, Zhang M, et al. The protective mechanism of human umbilical cord mesenchymal stem cell-derived exosomes against neutrophil extracellular trap-induced placental damage. Placenta. 2024;153:59–74. 10.1016/j.placenta.2024.05.136 38823320

[10] Smith S, Smith J, Jones K, Castillo A, Wiemann N, Howard A, et al. Placental ischemia during pregnancy induces hypertension, cerebral inflammation, and oxidative stress in dams postpartum. Hypertens Pregnancy. 2025;44(1):2454597. 10.1080/10641955.2025.2454597 PMC11849403 39885618

[11] Spaans F, de Vos P, Bakker WW, van Goor H, Faas MM. Danger signals from ATP and adenosine in pregnancy and preeclampsia. Hypertension. 2014;63(6):1154–60. 10.1161/HYPERTENSIONAHA.114.03240 24688119

[12] Panicucci C, Raffaghello L, Bruzzone S, Baratto S, Principi E, Minetti C, et al. eATP/P2X7R axis: an orchestrated pathway triggering inflammasome activation in muscle diseases. Int J Mol Sci. 2020;21(17). 10.3390/ijms21175963 PMC7504480 32825102

[13] Spaans F, Melgert BN, Chiang C, Borghuis T, Klok PA, de Vos P, et al. Extracellular ATP decreases trophoblast invasion, spiral artery remodeling and immune cells in the mesometrial triangle in pregnant rats. Placenta. 2014;35(8):587–95. 10.1016/j.placenta.2014.05.013 24953164

[14] Di Virgilio F, Vultaggio-Poma V, Falzoni S, Giuliani AL. Extracellular ATP: A powerful inflammatory mediator in the central nervous system. Neuropharmacology. 2023;224:109333. 10.1016/j.neuropharm.2022.109333 36400278

[15] Shen J, Liao B, Gong L, Li S, Zhao J, Yang H, et al. CD39 and CD73: biological functions, diseases and therapy. Mol Biomed. 2025;6(1):97. 10.1186/s43556-025-00345-9 PMC12586806 41188606

[16] Li X, Shen Y, Lang J, Wu J, Qian Z, Shen G, et al. CD39 Contributes to the Ability of Cell Invasion in Heterogeneity of Colorectal Cancer. J Cell Mol Med. 2025;29(5):e70486. 10.1111/jcmm.70486 PMC11886887 40052646

[17] McRae JL, Russell PA, Chia JS, Dwyer KM. Overexpression of CD39 protects in a mouse model of preeclampsia. Nephrol (Carlton). 2013;18(5):351–5. 10.1111/nep.12058 23600369

[18] Samudra AN, Dwyer KM, Selan C, Freddi S, Murray-Segal L, Nikpour M, et al. CD39 and CD73 activity are protective in a mouse model of antiphospholipid antibody-induced miscarriages. J Autoimmun. 2018;88:131–8. 10.1016/j.jaut.2017.10.009 29103803

[19] Duan L, Ma Y, Reisch B, Hadrovic E, Mach P, Kimmig R, et al. Alteration in sB7-H4 serum levels and placental biomarker expression after therapeutic plasma exchange in early-onset preeclampsia patients. Int J Mol Sci. 2024;25(20). 10.3390/ijms252011082 PMC11507903 39456867

[20] Leaños-Miranda A, Navarro-Romero CS, Sillas-Pardo LJ, Ramírez-Valenzuela KL, Isordia-Salas I, Jiménez-Trejo LM. Soluble Endoglin As a Marker for Preeclampsia, Its Severity, and the Occurrence of Adverse Outcomes. Hypertension. 2019;74(4):991–7. 10.1161/HYPERTENSIONAHA.119.13348 31446801

[21] Vicen M, Igreja Sá IC, Tripská K, Vitverová B, Najmanová I, Eissazadeh S, et al. Membrane and soluble endoglin role in cardiovascular and metabolic disorders related to metabolic syndrome. Cell Mol Life Sci. 2021;78(6):2405–18. 10.1007/s00018-020-03701-w PMC11072708 33185696

[22] Margioula-Siarkou G, Margioula-Siarkou C, Petousis S, Margaritis K, Vavoulidis E, Gullo G, et al. The role of endoglin and its soluble form in pathogenesis of preeclampsia. Mol Cell Biochem. 2022;477(2):479–91. 10.1007/s11010-021-04294-z 34783962

[23] Tyshchuk E, Grebenkina P, Krutetskaya I, Smirnov I, Stolbovaya A, Shashkova O, et al. Endoglin Regulates Intercellular Interactions between Trophoblast and Natural Killer Cells. J Evol Biochem Physiol. 2024;60(3):930–46.

[24] Peres RS, Donate PB, Talbot J, Cecilio NT, Lobo PR, Machado CC, et al. TGF-β signalling defect is linked to low CD39 expression on regulatory T cells and methotrexate resistance in rheumatoid arthritis. J Autoimmun. 2018;90:49–58. 10.1016/j.jaut.2018.01.004 29426578

[25] Pérez-Roque L, Núñez-Gómez E, Rodríguez-Barbero A, Bernabéu C, López-Novoa JM, Pericacho M. Pregnancy-induced high plasma levels of soluble endoglin in mice lead to preeclampsia symptoms and placental abnormalities. Int J Mol Sci. 2020;22(1). 10.3390/ijms22010165 PMC7795873 33375253

[26] Magee LA, Brown MA, Hall DR, Gupte S, Hennessy A, Karumanchi SA, et al. The 2021 International Society for the Study of Hypertension in Pregnancy classification, diagnosis & management recommendations for international practice. Pregnancy Hypertens. 2022;27:148–69. 10.1016/j.preghy.2021.09.008 35066406

[27] Guo Y, Zhu Y, Sun Y, Yang H. The preventive effect of low-dose aspirin in a PPAR-γ antagonist treated mouse model of preeclampsia. BMC Pregnancy Childbirth. 2022;22(1):606. 10.1186/s12884-022-04901-x PMC9338524 35906540

[28] Santulli G, Kansakar U, Varzideh F. Epidemiology and Pathophysiology of Preeclampsia: New Mechanistic Insights. Hypertension. 2025;82(5):800–3. 10.1161/HYPERTENSIONAHA.124.24117 PMC12007696 40238910

[29] Torres-Torres J, Espino YSS, Martinez-Portilla R, Borboa-Olivares H, Estrada-Gutierrez G, Acevedo-Gallegos S, et al. A narrative review on the pathophysiology of preeclampsia. Int J Mol Sci. 2024;25(14). 10.3390/ijms25147569 PMC11277207 39062815

[30] Das UN, Angiogenic. Antiangiogenic Molecules, and Bioactive Lipids in Preeclampsia. Am J Hypertens. 2017;30(9):864–70. 10.1093/ajh/hpx120 28830084

[31] Valbuena-Diez AC, Blanco FJ, Oujo B, Langa C, Gonzalez-Nuñez M, Llano E, et al. Oxysterol-induced soluble endoglin release and its involvement in hypertension. Circulation. 2012;126(22):2612–24. 10.1161/CIRCULATIONAHA.112.101261 23110859

[32] Shao X, Yu W, Yang Y, Wang F, Yu X, Wu H, et al. The mystery of the life tree: the placentas†. Biol Reprod. 2022;107(1):301–16. 10.1093/biolre/ioac095 35552600

[33] Staff AC. The two-stage placental model of preeclampsia: An update. J Reprod Immunol. 2019;134–135:1–10. 10.1016/j.jri.2019.07.004 31301487

[34] Burton GJ, Redman CW, Roberts JM, Moffett A. Pre-eclampsia: pathophysiology and clinical implications. BMJ. 2019;366:l2381. 10.1136/bmj.l2381 31307997

[35] Levine RJ, Lam C, Qian C, Yu KF, Maynard SE, Sachs BP, et al. Soluble endoglin and other circulating antiangiogenic factors in preeclampsia. N Engl J Med. 2006;355(10):992–1005. 10.1056/NEJMoa055352 16957146

[36] Horvat Mercnik M, Schliefsteiner C, Sanchez-Duffhues G, Wadsack C. TGFβ signalling: a nexus between inflammation, placental health and preeclampsia throughout pregnancy. Hum Reprod Update. 2024;30(4):442–71. 10.1093/humupd/dmae007 PMC11215164 38519450

[37] Lawera A, Tong Z, Thorikay M, Redgrave RE, Cai J, van Dinther M, et al. Role of soluble endoglin in BMP9 signaling. Proc Natl Acad Sci U S A. 2019;116(36):17800–8. 10.1073/pnas.1816661116 PMC6731690 31431534

[38] Cheifetz S, Bellón T, Calés C, Vera S, Bernabeu C, Massagué J, et al. Endoglin is a component of the transforming growth factor-beta receptor system in human endothelial cells. J Biol Chem. 1992;267(27):19027–30. 1326540

[39] Parhizkar F, Kiani A, Darzi S, Motavalli R, Noori Dolama F, Yousefzadeh Y, et al. The evaluation of CD39, CD73, and HIF-1 α expression besides their related miRNAs in PBMCs of women with recurrent pregnancy loss. J Reprod Immunol. 2023;156:103820. 10.1016/j.jri.2023.103820 36758470

[40] Yousefzadeh Y, Soltani-Zangbar MS, Kalafi L, Tarbiat A, Shahmohammadi Farid S, Aghebati-Maleki L, et al. Evaluation of CD39, CD73, HIF-1α, and their related miRNAs expression in decidua of preeclampsia cases compared to healthy pregnant women. Mol Biol Rep. 2022;49(11):10183–93. 10.1007/s11033-022-07887-z 36048381

[41] Fang F, Cao W, Mu Y, Okuyama H, Li L, Qiu J, et al. IL-4 prevents adenosine-mediated immunoregulation by inhibiting CD39 expression. JCI Insight. 2022;7(12). 10.1172/jci.insight.157509 PMC9309057 35730568

[42] Baek AE, Kanthi Y, Sutton NR, Liao H, Pinsky DJ. Regulation of ecto-apyrase CD39 (ENTPD1) expression by phosphodiesterase III (PDE3). Faseb j. 2013;27(11):4419–28. 10.1096/fj.13-234625 PMC3804755 23901069

[43] Harshe RP, Xie A, Vuerich M, Frank LA, Gromova B, Zhang H, et al. Endogenous antisense RNA curbs CD39 expression in Crohn’s disease. Nat Commun. 2020;11(1):5894. 10.1038/s41467-020-19692-y PMC7676266 33208731

[44] Zhao R, Qiao J, Zhang X, Zhao Y, Meng X, Sun D, et al. Toll-Like Receptor-Mediated Activation of CD39 Internalization in BMDCs Leads to Extracellular ATP Accumulation and Facilitates P2X7 Receptor Activation. Front Immunol. 2019;10:2524. 10.3389/fimmu.2019.02524 PMC6834529 31736956

[45] Yegutkin GG, Boison D. ATP and Adenosine Metabolism in Cancer: Exploitation for Therapeutic Gain. Pharmacol Rev. 2022;74(3):797–822. 10.1124/pharmrev.121.000528 PMC9553103 35738682

[46] Kennedy C. The P2Y/P2X divide: How it began. Biochem Pharmacol. 2021;187:114408. 10.1016/j.bcp.2021.114408 33444568

[47] Chen KY, Chan HC, Chan CM. Unveiling the P2X7 receptor: Exploring its mechanisms, pathogenic role in ocular diseases, and emerging therapeutic potential. Mol Aspects Med. 2025;105:101389. 10.1016/j.mam.2025.101389 40729957

[48] Savio LEB, de Andrade Mello P, Figliuolo VR, de Avelar Almeida TF, Santana PT, Oliveira SDS, et al. CD39 limits P2X7 receptor inflammatory signaling and attenuates sepsis-induced liver injury. J Hepatol. 2017;67(4):716–26. 10.1016/j.jhep.2017.05.021 PMC5875702 28554875

[49] Tariq M, Shaheen G, David M, Jahan S, Afsar T, Husain FM, et al. Evaluation of oxidative stress markers and the angiogenic factors in preeclampsia and associated features. BMC Pregnancy Childbirth. 2025;25(1):1006. 10.1186/s12884-025-08165-z PMC12492581 41039366

[50] Varejckova M, Gallardo-Vara E, Vicen M, Vitverova B, Fikrova P, Dolezelova E, et al. Soluble endoglin modulates the pro-inflammatory mediators NF-κB and IL-6 in cultured human endothelial cells. Life Sci. 2017;175:52–60. 10.1016/j.lfs.2017.03.014 28336397

[51] Wang J, Luo J, Rotili D, Mai A, Steegborn C, Xu S, et al. SIRT6 Protects Against Lipopolysaccharide-Induced Inflammation in Human Pulmonary Lung Microvascular Endothelial Cells. Inflammation. 2024;47(1):323–32. 10.1007/s10753-023-01911-5 37819455

[52] Procopciuc LM, Caracostea GV, Hangan AC, Lucaciu RL. Understanding Preeclampsia: Integrating Placental Dysfunction, Immune Dysregulation and microRNA-Mediated Epigenetic Regulation. Int J Mol Sci. 2026;27(10):4281. 10.3390/ijms27104281 PMC13207942 42196264

[53] Ma W, Gu Z, Lin Q, Cao M, Zhong J, Li X, et al. Integrative genetic and multi-omics analysis reveals the interleukin-6 receptor’s role in recurrent spontaneous abortion. Front Immunol. 2025;16–2025. 10.3389/fimmu.2025.1659251 PMC12528022 41112270

[54] Tang N, Wang H, He M, Geng N, Zhao X, Zhu X, et al. Mitochondria-mediated pyroptosis: anti-glioblastoma mechanism of the frankincense-derived compound β-boswellic acid. 3 Biotech. 2026;16(2):73. 10.1007/s13205-025-04691-x PMC12804556 41550484

[55] Hussain MS, Agrawal N, Ilma B, Nayak MMR, Kaur PP. Autophagy and Cellular Senescence in Alzheimer’s Disease: Key Drivers of Neurodegeneration. CNS Neurosci Ther. 2025;31(7):e70503. 10.1111/cns.70503 PMC12287388 40702750

[56] Barsoum IB, Renaud SJ, Graham CH. Glyceryl trinitrate inhibits hypoxia-induced release of soluble fms-like tyrosine kinase-1 and endoglin from placental tissues. Am J Pathol. 2011;178(6):2888–96. 10.1016/j.ajpath.2011.02.013 PMC3124015 21641407

[57] Cudmore M, Ahmad S, Al-Ani B, Fujisawa T, Coxall H, Chudasama K, et al. Negative regulation of soluble Flt-1 and soluble endoglin release by heme oxygenase-1. Circulation. 2007;115(13):1789–97. 10.1161/CIRCULATIONAHA.106.660134 17389265

[58] Yao YL, Xu Z, Xiao R, Li EH, Zhang YJ, Zhou LY, et al. Elevated Neutrophil, Lymphocyte, and Platelet Counts as Early Biomarkers of Preeclampsia Risk: A Retrospective Cohort Study. Am J Reprod Immunol. 2025;94(2):e70137. 10.1111/aji.70137 40736482

