# Supplementary material for: Soluble endoglin induces trophoblast apoptosis and dysfunction via the CD39-ATP signaling axis: a potential pathogenic mechanism of preeclampsia
Source: J Transl Med. 2026 Aug 10;24:1400. doi: 10.1186/s12967-026-08759-x (PMC13741447; doi:10.1186/s12967-026-08759-x)
Supplement: Supplementary file 1 [file 12967_2026_8759_MOESM1_ESM.doc]

**Supplementary Material**

**Section 1. Baseline characteristics and related data**

Among the 10 enrolled patients with PE, one met the criteria for early-onset PE and nine for late-onset PE. Eight cases were categorized as mild PE, and two as severe PE. The therapeutic regimen for all patients was restricted to oral antihypertensive drugs combined with intravenous magnesium sulfate.

Detailed clinical and laboratory data are summarized in Table S1. Fetal growth restriction (FGR) was defined when the fetal weight below the 10th percentile for gestational age. Data were presented as mean ± SEM. Continuous variables were compared using the independent t-test, while nonparametric tests were applied to parity, prenatal systolic blood pressure and AST due to heterogeneity of variance. Categorical variables were analyzed via the chi-square test.

| Table S1. Clinical and demographic data for healthy controls and patients with preeclampsia who provided samples in this study | | | |
| --- | --- | --- | --- |
| Variable | Healthy control (n=10) | Preeclampsia (n=10) | P value |
| Maternal age (years) | 31.40±4.22 | 33.80±3.97 | 0.207 |
| Pre-pregnancy BMI (kg/m2) | 23.03±3.38 | 25.96±3.52 | 0.073 |
| Conception mode |  |  |  |
| Spontaneous conception, n(%) | 1 (10%) | 1 (10%) | 1 |
| Assisted reproductive technology, n(%) | 9 (90%) | 9 (90%) |  |
| Parity(n) | 1.20±0.42 | 1.30±0.48 | 0.739 |
| Labor status |  |  |  |
| In labor, n(%) | 7 (70%) | 2 (20%) | 0.07 |
| Not in labor, n(%) | 3 (30%) | 8 (80%) |  |
| Gestational age at delivery (wk) | 38.86 ± 1.12 | 37.72 ± 1.62 | 0.083 |
| Gestational SBP before 20week gestation (mm Hg) | 115.60 ± 10.49 | 116.40 ± 6.72 | 0.841 |
| Gestational DBP before 20 week gestation (mm Hg) | 72.90 ± 5.55 | 74.60 ± 4.55 | 0.463 |
| Gestational SBP, pre-delivery (mm Hg) | 118.80 ± 3.88 | 133.90 ± 13.93 | 0.015* |
| Gestational DBP, pre-delivery (mm Hg) | 74.40 ± 5.80 | 81.00 ± 7.18 | 0.036* |
| 24-hour urinary protein (mg) | / | 1285.60±1186.18 | / |
| ALT (U/L) | 11.07±2.73 | 10.66±4.69 | 0.814 |
| AST (U/L) | 36.95±56.71 | 16.34±5.28 | 0.247 |
| Platelet count (×10^9^/L) | 187.10±34.94 | 166.80±25.53 | 0.155 |
| Preterm birth |  |  |  |
| Yes, n(%) | 1 (10%) | 1 (10%) | 1 |
| No, n(%) | 9 (90%) | 9 (90%) |  |
| Fetal growth restriction |  |  |  |
| Absent, n (%) | 0 (0%) | 1 (10%) | 0.474 |
| Present, n (%) | 10 (100%) | 9 (90%) |  |
| Delivery mode |  |  |  |
| Cesarean section, n(%) | 3 (30%) | 8 (80%) | 0.07 |
| Vaginal delivery, n(%) | 7 (70%) | 2 (20%) |  |
| Fetal sex |  |  |  |
| Male, n(%) | 7 (70%) | 6 (60%) | 1 |
| Female, n(%) | 3 (30%) | 4 (40%) |  |
| Fetal birth weight (g) | 3469.00±350.19 | 3124.00±585.50 | 0.127 |
| BMI, body mass index; DBP/SBP, diastolic/systolic blood pressure; ALT, alanine aminotransferase; AST, aspartate aminotransferase. *p<0.05. | | | |

**Section 2. Experimental reagents: antibodies and primers**

**Table S2. List of primary, secondary antibodies for immunofluorescence in this study**

| **Primary antibody** | **Host** | **Reactivity** | **Company** | **Cat. No.** |
| --- | --- | --- | --- | --- |
| CD39 | Rabbit | Mouse | DiagBio | DB12858 |
| P2X7R | Rabbit | Human, mouse | Proteintech | 28207-1-AP |
| CK7 | Mouse | Human, mouse | ZenBio | 250022 |
| CD39 | Rabbit | Human | TengKe Bio-TECH | TKAB3087 |
| Cleaved caspase-3 | Rabbit | Human, mouse | Affinity Biosciences | AF7022 |
| Bcl-2 | Rabbit | Human | TengKe Bio-TECH | TKAB21486 |
| Ki-67 | Rabbit | Human | Sigma | SAB5700770 |
| **Secondary antibody** | **Host** | | **Company** | **Cat. No.** |
| Alexa Fluor 488 | Donkey anti rabbit | | Invitrogen | R37118 |
| Cy3 | Goat anti mouse | | Invitrogen | A10521 |
| Cy3 | Goat anti rabbit | | Invitrogen | A10520 |

**Table S3. List of primary, secondary antibodies for immunohistochemistry in this study**

| **Primary antibody** | **Company** | **Cat. No.** |
| --- | --- | --- |
| Bax (2Q11) Rabbit Monoclonal Antibody | Biodragon | RM1267 |
| Bcl-2(P65) Rabbit pAb | TengKe Bio-TECH | TKAB21486 |
| Cleaved-Caspase 3 (Asp175), p17 Antibody | Affinity Biosciences | AF7022 |
| **Secondary antibody** | **Company** | **Cat. No.** |
| DAB stanning（MaxVision III mouse/rabbit） | FUZHOU MAIXIN BIOTECH, LTD | KIT-5230 |

**Table S4. List of primary, secondary antibodies and dilution factors for western blotting in this study**

| **Primary antibody** | | | |
| --- | --- | --- | --- |
| Name | Company | Cat. No. | Concentration |
| Bax (2Q11) Rabbit Monoclonal Antibody | Biodragon | RM1267 | 1:1000 |
| p53 Rabbit Polyclonal Antibody | Biodragon | BD-PT3528 | 1:1000 |
| Caspase 3 (4I7) Rabbit Monoclonal Antibody | Biodragon | RM7070 | 1:1000 |
| Cleaved-Caspase 3 (Asp175), p17 Antibody | Affinity Biosciences | AF7022 | 1:750 |
| Bcl2(P65) Rabbit pAb | TengKe Bio-TECH | TKAB21486 | 1:1000 |
| CD39 (6E14) Rabbit Monoclonal Antibody | Biodragon | RM3393 | 1:750 |
| Smad3 (12G9) Rabbit Monoclonal Antibody | Biodragon | RM1243 | 1:1000 |
| Smad3 (Phospho Ser423/425) (12O16) Rabbit Monoclonal Antibody | Biodragon | RM1186 | 1:1000 |
| Smad2 (19V12) Rabbit Monoclonal Antibody | Biodragon | RM4123 | 1:1000 |
| Smad2 (Phospho Ser465/467) Rabbit Polyclonal Antibody | Biodragon | BD-PP1500 | 1:1000 |
| GAPDH (2B8) Mouse mAb (HRP) | Immunoway | YM2054 | 1:5000 |
| Tubulin β mAb-HRP | TengKe Bio-TECH | TKAB0424 | 1:5000 |
| **Secondary antibody** | | | |
| Name | Company | Cat. No. | Concentration |
| HRP-Goat anti rabbit IgG(H+L) | Biodragon | BF03008 | 1:10000 |

**Table S5. List of primers for qPCR in this study**

| Gene | Primer Sequence (5’ → 3’) |
| --- | --- |
| CD39 | for:ATCCTTGGCTTCTCCTCTAT  rev: ACCCTGCATTCTTCTACTTG |
| GAPDH | Human GAPDH qPCR Primer Pair（QH0009L,Beyotime，Shanghai, China） |

**Section 3. Supplementary experimental methods**

****Single-cell RNA Sequencing Data Processing and Quality Control****

**Single-cell RNA sequencing data from four placental tissue samples were included in this study, consisting of two control samples (Ctrl: GSM5261695_C1, GSM5261696_C2) and two preeclampsia (PE) samples (PE: GSM5261699_P1 and GSM5261700_P2). During data preprocessing, a statistical approach based on the Median Absolute Deviation (MAD) was applied to the raw gene expression matrix for rigorous quality control (QC) to identify and exclude outlier cells. Specifically, cells with abnormal total UMI counts (log1p_total_counts), detected genes (log1p_n_genes_by_counts), or the proportion of counts from the top 20 highly expressed genes (pct_counts_in_top_20_genes) exceeding 5 the MAD were removed. Cells with mitochondrial gene expression proportions (pct_counts_mt) exceeding 3 × MAD or an absolute mitochondrial proportion of >8% were excluded. Violin and scatter plots were generated to evaluate the data quality and filtering efficiency. After quality control, 22,532 cells were retained for downstream analyses, including 10,648 cells from the control group and 11,884 cells from the PE group. To minimize batch effects among individual samples, data integration and batch correction were performed using scVI, with the sample identity specified as the batch covariate. The latent representation generated by scVI was used for all downstream analyses, including dimensionality reduction, clustering and visualization.**

****Dimensionality Reduction, Clustering, and Cell Type Annotation****

**The batch-corrected latent embedding generated by scVI was used for downstream analysis. Principal Component Analysis (PCA) was first performed, followed by Uniform Manifold Approximation and Projection (UMAP) for nonlinear dimensionality reduction and visualization. Graph-based clustering was performed using the Leiden algorithm with a resolution of 0.6, resulting in the identification of 20 distinct cell clusters (clusters 0–19).**

**Cell type annotation was performed according to the expression profiles of cluster-specific marker genes, leading to the identification of multiple placental cell populations, including B_Cells, Epithelial_Cells, Extravillous Trophoblast (EVT)Precursors, EVT, Hofbauer_Cells, Macrophages, MAST_Cells, Neutrophils, Natural Killer Cells (NK_Cells), syncytiotrophoblasts (SCT), Stromal_Cells, T_Cells, and Villous Cytotrophoblasts (VCT).**

****Differentially Expressed Genes and Functional Enrichment Analysis****

**To investigate transcriptomic alterations associated with preeclampsia, differential gene expression analysis was performed independently for each annotated cell type by comparing PE and control cells. Differentially expressed genes (DEGs) were identified using a threshold of log fold change (logFC) > 1 and an adjusted P-value < 0.05. Multiple testing correction was performed using the false discovery rate (FDR) method. Gene Ontology (GO) and Kyoto Encyclopedia of Genes and Genomes (KEGG) enrichment analyses were subsequently performed separately for upregulated and downregulated DEGs to characterize the biological processes and signaling pathways associated with PE.**

****AUCell Gene Set Scoring and Expression Visualization****

**To evaluate functional alterations across different placental cell populations, pathway activity was quantified at the single-cell level using the AUCell algorithm, which calculates enrichment scores based on ranked gene expression within individual cells. AUCell was applied to apoptosis- and ATP-related gene sets to assess the cellular survival status and energy metabolism under physiological and pathological conditions. AUCell scores were compared between the control and PE groups within each cell type, and pathways were prioritized based on P < 0.05. The expression patterns of representative molecules, including ENTPD1 (CD39) and SMAD family members, were visualized to validate the pathway-associated molecular changes.**.

**Section 4. Supplementary results**

**Figure S1**

Figure S1. Validation of placenta-specific mENG overexpression in pregnant mice administered with AAV6-mENG. AAV6-hrGFP: Pregnant mice injected with AAV6-hrGFP served as the control group (n=8). AAV6-mENG: Pregnant mice received an equal volume of AAV6-mENG for mENG overexpression (n=8). (A-B) Representative western blot images and analysis of mENG protein expression in liver, kidney, lung and placenta tissues from two groups; GAPDH was used as the internal reference; (C) Representative immunohistochemical staining of mENG in liver, kidney, lung and placental sections of two groups; (D) ELISA quantification of circulating sENG concentration in peripheral serum of pregnant mice from the two groups.

Statistical comparisons were performed using two-way ANOVA (B) and unpaired Student’s t-test (C). Data are presented as mean ± SEM. ****P < 0.0001.

**FigureS2**

Figure S2. Quality control assessment of single-cell RNA-sequencing data. (A-H) Violin plots showing the distribution of the number of genes (n_genes_by_counts), total UMI counts (total_counts), and mitochondrial gene percentage (pct_counts_mt) in individual cells from samples GSM5261695_C1, GSM5261696_C2, GSM5261699_P1, and GSM5261700_P2.

**Figure S3**

Figure S3. Single-cell transcriptome profiling and cell type identification in control and preeclampsia samples. (A) UMAP visualization of all cells from control (Ctrl) and preeclampsia (PE) samples, showing the global cellular distribution; (B) Cell clustering results using the Leiden algorithm at a resolution of 0.6, identifying 20 distinct cell clusters; (C) UMAP plot of cell type annotation based on canonical marker genes, with 12 major cell types identified; (D) Bar chart quantifying the number of cells per cell type in the control and PE groups; (E) UMAP visualization of the spatial distribution of different cell types in control samples; (F) UMAP visualization of the spatial distribution of different cell types in PE samples; (G) Integrated analysis of cell‑type‑specific marker genes and functional enrichment, including violin plots of marker gene expression across cell types (left), scaled expression heatmap of marker genes (middle), and bar plots of GO‑BP enrichment analysis for the corresponding cell types (right).

**Figure S4**

Figure S4. Functional enrichment analysis and gene expression patterns in control and preeclampsia samples. (A) Functional enrichment analysis of differentially expressed genes, including Gene Ontology (GO) terms for biological process (BP), cellular component (CC), and molecular function (MF), as well as KEGG and Reactome pathway enrichment results; (B) Violin plots comparing the pathway scores of “apoptosis-induced DNA fragmentation” in each cell type between control (Ctrl) and preeclampsia (PE) groups; (C) Violin plots comparing the pathway scores of “ATP biosynthetic process (GO:0006754)’ in each cell type between control (Ctrl) and preeclampsia (PE) groups; (D-E) Bubble plots showing the expression of key genes ENTPD1 (D) and SMAD2/SMAD3 (E) across cell types. Bubble size indicates the proportion of positive cells, and color intensity represents the average expression level.

**Figure S5**

Figure S5. The change of CD39 expression and eATP level after the application of CD39 inhibitor ARL67156 in HTR-8/SVneo. (A) CD39 immunofluorescence staining (red) in ARL67156-treated HTR-8/SVneo (0, 10, 20 μM); nuclei counterstained with DAPI.(blue); (B) ATP level in the culture supernatant of ARL67156-treated HTR-8/SVneo (0, 10, 20 μM).

The significance of all data was determined using one-way ANOVA, followed by Dunnett’s post-hoc test. Data were presented as the mean±SEM of three independent experiments. *P < 0.05, ** P<0.01,***P < 0.001,****P < 0.0001.

**Figure S6**

Figure S6. Functional changes in HTR-8/SVneo after BzATP treatment with or without the P2X7R inhibitor A-740003. (A) Ki67 immunofluorescence staining (green) in BzATP(with or without A-740003)-treated HTR-8/SVneo; nuclei counterstained with DAPI.(blue); (B)Caspase-3 activity assay for each group.

**Figure S7**

Figure S7. Dose-dependent induction of apoptosis and disruption of CD39-ATP axis by sENG in BeWo trophoblast cells. (A-B) Annexin V-FITC/PI flow cytometry and quantitative statistics of total apoptosis rate in control, 40 ng/mL and 80 ng/mL sENG-treated BeWo cells. (C) Representative immunofluorescence images of CD39 (green, DAPI for nuclei, scale bar:100 μm) after sENG treatment; (D) Quantitative measurement of supernatant ATP concentration after sENG treatment.

The significance of all data was determined using one-way ANOVA, followed by Dunnett’s post-hoc test. Data were presented as the mean±SEM of three independent experiments. *P < 0.05, ** P<0.01.
