# Supplementary material for: Soluble endoglin induces trophoblast apoptosis and dysfunction via the CD39-ATP signaling axis: a potential pathogenic mechanism of preeclampsia
Source: J Transl Med. 2026 Aug 10;24:1400. doi: 10.1186/s12967-026-08759-x (PMC13741447; doi:10.1186/s12967-026-08759-x)
Supplement: Supplementary file 2 [file 12967_2026_8759_MOESM2_ESM.doc]

**The original images of blots**

**From submitted paper Yu Wang et al.**

**The protein ladder (TengKe Bio-TECH, TKWB0105-250) was used in all figures.**

**The original blot images for Figure 2**

(A) p-Smad2 Blot edge with marker	(B) Smad2 Blot edge with marker

(C) GAPDH Blot edge with marker

(D) p-Smad3Blot edge with marker	(E) Smad3 Blot edge with marker

(F) GAPDH Blot edge with marker

(G) CD39 Blot edge with marker	(H) GAPDH Blot edge with marker

**The original blot images for Figure 3**

(A) BAX Blot edge with marker	(B) β-tubulin Blot edge with marker

(C) Bcl-2 Blot edge with marker	(D) β-tubulin Blot edge with marker

(E) Caspase3 and Actived-Caspase3 Blots edge with marker (F) β-tubulin Blot edge with marker

(G) p53 Blot edge with marker	(H) GADPH Blot edge with marker

**The original blot images for Figure 4**

(A) p-Smad2 Blot	(B) Smad2 Blot	(C) GAPDH Blot

(D) p-Smad3 Blot	(E) Smad3 Blot	(F) GAPDH Blot

(g) p-Smad3 Blot	(H) GAPDH Blot

**The original blot images for Figure 5**

(A) Caspase3 Blot	(B) β-tubulin Blot

(C) BAX Blot	(D) β-tubulin Blot

(E) Bcl-2 Blot	(F) β-tubulin Blot

**The original blot images for Figure 7**

(A) CD39 Blot	(B)GAPDH Blot

(C) CD39 Blot	(D)GAPDH Blot

**The original blot images for Figure 8**

(A) BAX Blot	(B) Caspase3 Blot

(C) β-tubulin Blot

(D) Bcl-2 Blot	(E) β-tubulin Blot

(F) Bcl-2 Blot	(G) β-tubulin Blot

(H) p-Smad2 Blot	(I) Smad2 Blot

(J) GAPDH Blot

(K) p-Smad3 Blot	(L) Smad3 Blot

(M) GAPDH Blot

**The original blot images for Figure S1**

(A) ENG Blot	(B)GAPDH Blot
